# The Amplitude Modulation Structure of Japanese Infant- and Child-Directed Speech: Longitudinal Data Reveal Universal Acoustic Physical Structures That Accommodate Both Syllabic and Moraic Timing

**DOI:** 10.1162/NOL.a.226

**Published:** 2026-03-26

**Authors:** Tatsuya Daikoku, Usha Goswami

**Affiliations:** Centre for Neuroscience in Education, University of Cambridge, Cambridge, UK; Graduate School of Information Science and Technology, The University of Tokyo, Tokyo, Japan

**Keywords:** amplitude modulation, phonological hierarchy, rhythm, speech

## Abstract

Infant-directed speech (IDS) is highly rhythmic, and in European languages, it is dominated by patterns of amplitude modulation (AM) peaking at ∼2 Hz (reflecting prosody) and ∼5 Hz (reflecting individual syllables). The rhythm structure of spoken Japanese is thought to differ from European stress-timed and syllable-timed languages, depending on moraic units (∼10 Hz) comprising any onset phoneme and vowel phonemes within a syllable, PA-N-DA. As the infant brain must be prepared to acquire any human language, initial speech encoding is likely to utilize language-universal physical acoustic structures in speech. These physical structures are, however, probabilistic and may thereby simultaneously accommodate language-specific structures like morae. Here, a language-blind computational model of linguistic rhythm based on the amplitude envelope (AE) is used to compute the physical acoustic stimulus characteristics for Japanese. Using ∼18,000 samples of natural IDS and child-directed speech (CDS) recorded longitudinally over the ages 0–5 years, the data show that the temporal modulation patterns that characterize the AE of Japanese are similar to those found for stress-timed and syllable-timed European languages. However, the AM band corresponding to the syllabic level in CDS/IDS in European languages (∼2–12 Hz) was elongated in Japanese (2.5–17 Hz), possibly accommodating the faster modulation peaks reflecting morae. Furthermore, the phase synchronization ratios between the two slowest AM bands were as likely to be 1:3 as 1:2, differing from European languages where 1:2 ratios (delivering the perceptual experience of a temporally regular beat) are dominant. Accordingly, the amplitude-driven physical acoustic structures important for cortical speech tracking flexibly accommodate both universality and specificity.

## INTRODUCTION

How infants, born without understanding or producing words, acquire spoken language is a long-standing research puzzle. A central piece of this linguistic puzzle is infant-directed speech (IDS)—the distinctive, melodic form of speech that caregivers instinctively use when speaking to babies. From their first moments of birth, infants are surrounded by IDS, and the rich auditory and rhythmic cues provided by IDS are thought to guide their earliest steps into language development. Caregivers unconsciously use IDS when interacting with their infants, and IDS has a number of defining characteristics including a simplified grammatical structure (Soderstrom, 2007), an exaggerated rhythmic structure (Leong et al., 2017), heightened pitch and exaggerated pitch range (Fernald & Simon, 1984; Fernald et al., 1989), and hyperarticulation of vowels (Kuhl, 2000, 2004; Kuhl et al., 1997). Yet, despite decades of research, much remains unknown concerning the neural and perceptual mechanisms that infants use to profit from the special characteristics of IDS to learn language.

In the current report, we adopt an oscillatory acoustic framework derived from recent research on the neural mechanisms of adult speech encoding and investigate whether early language acquisition may be grounded in the internalization of the acoustic rhythmic structures that exist across different languages. We use this neurally informed perspective to motivate speech modeling of the amplitude envelope (AE) of Japanese IDS and child-directed speech (CDS). The AE is the slow-varying energy profile of the acoustic waveform that is known to be tracked by cortical oscillations (Ahissar et al., 2001; Giraud & Poeppel, 2012) and captures variations in signal intensity (amplitude modulation, AM) over time (Houtgast & Steeneken, 1985). The AE of speech in different languages shows a highly consistent modulation structure, with adult-directed speech (ADS) showing a modulation peak at ∼5 Hz, thought to indicate syllabic structure (Ding et al., 2017; Varnet et al., 2017). However, AE-based speech modeling (following methods for demodulation pioneered by Turner, 2010; Turner & Sahani, 2011) has shown that there is systematic AM structure nested in the speech AE, which depends on the phase relations between peaks and troughs in different AM bands (Leong & Goswami, 2015). This AM structure both contributes to the perceptual experience of speech rhythm (whether a syllable is heard as stressed or unstressed; Leong et al., 2014) and enables the parsing of linguistic units such as syllables from the continuous signal (Leong & Goswami, 2015).

Key features of this AE modeling are the discovery of three systematic and relatively broad bandings of AMs in the AE of IDS and CDS of English, with center frequencies (modulation peaks) at ∼2, ∼4/5, and ∼20 Hz (Leong & Goswami, 2015; Leong et al., 2017). If one AM cycle at each temporal rate is assumed to match a linguistic unit, then for deliberately rhythmic speech the modeling can find the units of English stressed syllables, syllables and onset–rime units with > 90% accuracy (Leong & Goswami, 2015). These three broad bandings of AMs are hierarchically nested from slower to faster bands and are connected by phase relations. The phase alignment between the two slower AM bandings oscillates in a predominantly 1:2 ratio in English IDS and CDS, thereby yielding the perception of a regular rhythmic beat. Furthermore, at least for European languages, the AE-based modeling finds that these physical acoustic structures are consistent across CDS and ADS for languages with both stress-timed and syllable-timed rhythms (Araújo et al., 2018; Perez-Navarro et al., 2022; for deliberately rhythmic ADS [metrical poetry] in French and German, see Daikoku et al., 2024). Regarding language acquisition, the modeling has shown high similarity between Spanish and English regarding the acoustic physical structures of CDS, even though Spanish is syllable-timed and English is stress-timed (Perez-Navarro et al., 2022). Indeed, the phase alignment between the two slower AM bandings oscillates in a predominantly 1:2 ratio in both English and Spanish CDS, thereby yielding the perception of a regular rhythmic beat. Furthermore, the degree of phase alignment between the two slower AM bandings in English IDS and Spanish CDS is notably significantly greater than in English and Spanish ADS, resulting in IDS and CDS having a very perceptually salient rhythmic “beat” structure (Leong et al., 2017; Perez-Navarro et al., 2022). Whether Japanese IDS and CDS would exhibit a similar “beat” structure is currently untested.

The AM-based speech modeling of the AE of IDS is matched by recent infant studies of cortical tracking, which show that the infant brain can track the overall speech AE very successfully (Jessen et al., 2019; Kalashnikova et al., 2018; Nguyen et al., 2023). Regarding the AM bandings nested in English IDS that peak at ∼2 and ∼4/5 Hz, the Cambridge UK BabyRhythm study showed that the AM information in these individual bands is successfully encoded by neuroelectric oscillations operating at matching rhythmic rates, delta-band networks (0.5–4 Hz) and theta-band networks (4–8 Hz; Attaheri et al., 2022, 2024) from 4 months of age. Note that each electroencephalography band has a range of operating frequencies, indicating that neuronal oscillations are not temporally precise (Haegens et al., 2014). Rather, these cortical networks operate over a range of frequencies because temporal variability provides functional advantages, such as allowing the system to adapt to changing conditions or to increase the capacity for coding information despite temporal variation in that information (Keitel et al., 2025). It follows that the AM ranges characterizing different languages could be expected to show similar temporal variability if units salient to the linguistic structure of those languages occur at varying peak rates. Temporal Sampling theory argues that acoustic AM-driven rhythmic structures serve as a foundational perceptual scaffold for language acquisition (Goswami, 2022). Accordingly, the AE of IDS across languages might be expected to share similar AM structure, but with some temporal variability. Specifically, AE-based modeling should reveal broadly matching core AM bandings in IDS in all languages, connected by similar phase relations. However, these physical acoustic structures may also vary in the ranges of their operating frequencies in order to accommodate linguistic variability in specific temporal components that contribute to the perceptual experience of speech rhythm.

Therefore, while similar AM bandings should be found in IDS across languages, there may be variability regarding both the temporal range of each AM banding and of the modulation peak in each band, enabling the nascent speech processing system to encompass both universality and specificity. There may also be variability in the nature of the phase relations between AM bands. A good test of this hypothesis is IDS in Japanese. Japanese ADS is recognized for employing a mora-timed rhythmic structure, which sets it apart from the stress-timed rhythms of languages like English and German and the syllable-timed rhythms of languages like Portuguese, French, and Spanish (Cutler & Otake, 1994). Morae serve as fundamental perceptual and acoustic units for Japanese adults, influencing speech segmentation, lexical access, and overall language processing (Cutler, 2012). This rhythmic typology is thought to reflect a temporal organization of units occurring at frequencies around 8–10 Hz (Han, 1994; Warner & Arai, 2001). Mora-timed languages, which also include Gilbertese, Slovak, and Ganda, are unique in using morae—subsyllabic units with uniform temporal duration—as the foundational timing unit in ADS. For example, a word like PANDA would be segmented into the morae PA-N-DA rather than the syllables PAN-DA, with the vowel tied to the onset phoneme rather than to the coda (see Peter et al., 2022).

Prior research in Japanese IDS has similarly highlighted some unique linguistic characteristics. For example, while confirming that IDS in Japanese also uses heightened pitch and an exaggerated pitch range (as does IDS in European languages), Mazuka et al. (2015) demonstrated that intonation in Japanese IDS differs from patterns observed in English IDS. Intonation is exaggerated in ways consistent with the Japanese language's specific prosodic structure, thus exaggeration is found only at the boundary of prosodic phrases (rather than on any stressed syllable, as in English IDS). Tajima et al. (2013) found that morae duration in Japanese IDS was quite variable, although non–consonant–vowel morae (such as moraic nasals that characterize Japanese rhythm) occurred more frequently in IDS than in ADS. Indeed, Yoshida et al. (2010) reported that Japanese infants aged 7–8 months did not appear sensitive to grouping on the basis of durational cues, showing no preference between iambic versus trochaic grouping. Given the shared biophysical and neurophysiological constraints on speakers, Japanese IDS (and CDS) may exhibit matching hierarchical AM bandings as found in IDS and CDS in stress-timed (e.g., English) and syllable-timed (e.g., Spanish) languages but may alter the phase relations between the bands in order to accommodate moraic timing.

It should be noted that neonates have heard sufficient acoustic rhythmic information from within the womb to be able to discriminate between syllable-timed and stress-timed languages at birth (Mehler et al., 1988; Nazzi et al., 1998). This suggests that some spectrotemporal features contributing to rhythmic structure must vary by language. However, these features may not be primarily AM-based. To investigate these questions, here we apply the same AE-based modeling previously applied to English and Spanish IDS and CDS (Leong & Goswami, 2015; Leong et al., 2017; Perez-Navarro et al., 2022) to IDS and CDS in Japanese. Our key prediction is that Japanese IDS should share the universal hierarchical AM structures suited to cortical speech tracking that are found in stress-timed and syllable-timed languages. An open question is whether the three AM bandings will show similar center frequencies, similar temporal boundaries, and similar phase relations as found in European stress-timed and syllable-timed languages.

Previous research has also demonstrated that IDS and CDS are characterized by a slower tempo relative to ADS (Fernald et al., 1989; Grieser & Kuhl, 1988; Leong & Goswami, 2015; Perez-Navarro et al., 2022). This slower tempo enhances lower-frequency rhythmic modulations in the speech signal, which in turn may enhance neural entrainment by aligning with the intrinsic oscillatory dynamics of the infant brain (Nguyen et al., 2023). Accordingly, such tempo differences may contribute to the dominant AM bands observed in IDS/CDS across languages, including Japanese. On a language-universal hypothesis, however, stronger phase synchronization between the two slowest bands of AMs in Japanese IDS would still be anticipated in comparison to Japanese ADS, with a dominant integer ratio of 1:2. This 1:2 synchronization implies that one complete cycle of the slower AM band aligns with every two cycles of the faster AM band, a temporal structure known to enhance the perceptual experience of a regular metronome-like “beat” (Daikoku & Goswami, 2022; Leong et al., 2017). Perceptually, such integer ratios are thought to support the emergence of metrical grouping, providing the listener with a stable landmark beat or sense of rhythm to scaffold further linguistic learning. Given that Japanese has moraic as well as syllabic rhythms, the integer ratio of 1:2 may not be as dominant as found in English and Spanish.

In summary, the current study analyzed the acoustic structure of Japanese ADS, CDS, and IDS from a language-blind AE-perspective. The longitudinal database employed here comprised naturalistic speech recordings from three families, six parents, and five children. The speech interactions were recorded intermittently over a period of up to 5 years from the time of each infant’s birth (https://doi.org/10.32130/src.INFANT; Amano et al., 2008). By utilizing a longitudinal corpus previously used to demonstrate exaggerated pitch (Amano et al., 2006), here we provide complementary analyses of the acoustic structure of the AE of Japanese IDS/CDS relevant to cortical speech encoding (Giraud & Poeppel, 2012; Gross et al., 2013). Our acoustic modeling approach focuses on the AM hierarchy of sound waveforms below approximately 40 Hz, which characterize the speech signal as processed by human listeners (Moore, 2013). These different rates of AM arise from the coordinated movements of the vocal folds, tongue, vocal tract, mouth, and lips (Greenberg et al., 2003), which collectively generate a varying AE underpinning the perception of speech rhythm (Greenberg, 2006). Indeed, these motor movements exhibit a high degree of temporal consistency across languages (Poeppel & Assaneo, 2020).

Our modeling employed the Spectral-Amplitude Modulation Phase Hierarchy (S-AMPH) approach (Leong, 2012; Leong & Goswami, 2015), a computational framework based on demodulation (Turner, 2010; Turner & Sahani, 2011) designed to analyze the AM structure of the AE by isolating AM characteristics from frequency modulation components. The S-AMPH model provides a low-dimensional representation of the speech signal, capturing its dominant spectral (acoustic frequency, including pitch and formants) and temporal (oscillatory rate and speech rhythm) modulation patterns. This method enables the identification of predominant timescales of rhythmic modulation within Japanese speech. Furthermore, prior S-AMPH studies have demonstrated that IDS and music provide more rhythmically structured input than conversational ADS, as evidenced by stronger phase relationships between the two slowest AM bands and a prominent 1:2 ratio as regard their phase synchronization index (PSI; Daikoku & Goswami, 2022; Leong et al., 2017; Perez-Navarro et al., 2022). On a language-universal hypothesis, the phase relations between the slower AM bandings in Japanese IDS/CDS and Japanese ADS might be expected to show similar patterns.

Building on previous S-AMPH modeling in other languages, we thus hypothesize that the AM bands and their boundaries—defining the temporal modulation structure of spoken Japanese—will closely align with the AM bands identified in other languages. We also predict a comparable AM phase relationship pattern for Japanese IDS/CDS as found in these languages, with the two slowest AM bands showing a higher PSI in IDS/CDS than in ADS. A PSI of 1:2 would signal the presence of a distinct rhythmic beat, supporting the universality of rhythmic temporal structures based on phase relations in spoken language. The absence of a 1:2 PSI in Japanese would support the existence of a fundamentally different temporal rhythmic organization.

## MATERIALS AND METHODS

### Participants

This study utilized a longitudinal database of spontaneous speech recorded within the home environment from three families, comprising a total of five young children and their parents. Recordings spanned approximately 5 years, beginning shortly after each child's birth. Specifically, recordings occurred between 1988 and 2000, capturing developmental periods from infancy through early childhood (detailed recording periods provided in Table 1). The participant group included two boys and three girls. Families were structured as follows: Child A was a member of Family 1 and had a brother 10 years older; however, the brother was not included in the recordings. Children B and E were members of Family 2 and were a brother–sister pair. Children C and D were members of Family 3 and were sisters. All children were raised in urban areas speaking predominantly standard Japanese, minimizing regional dialect influence. Both parents and children were healthy with no reported abnormalities in speech perception or production. Additional detailed demographic data, such as precise ages during recordings, mean lengths, and dates for each speech sample, are summarized in Table 1 and provided comprehensively in an Excel file available on Open Science Framework (OSF; https://osf.io/n3upf/?view_only=9744b797056648cd80c7ec8f9732e093). Readers interested in more detailed original data should refer to Amano et al. (2008; https://doi.org/10.32130/src.INFANT). The present study involved only secondary analysis of an existing publicly available corpus (INFANT corpus; Amano et al., 2008). No new data collection or interaction with human participants was conducted. All original data were collected in accordance with prevailing ethical guidelines at the time of recording and with informed consent from participants.

**Table 1.** Information of the speech sample.

ID	Sex	At birth			Recording periods	Mean length (s ± *SD*)			
		Birth day	Height (mm)	Weight (g)		ADSm	ADSf	CDSm	CDSf
A	M	1988/4	510	3,450	1988/5∼1990/10	10.7 (±0.14)	10.9 (±0.22)	10.3 (±0.03)	10.4 (±0.15)
B	M	1990/5	480	3,250	1990/6∼1994/11	11.2 (±0.19)	11.1 (±0.17)	10.5 (±0.03)	10.4 (±0.03)
C	F	1991/6	495	3,440	1991/6∼1996/6	11.8 (±0.34)	10.8 (±0.29)	10.2 (±0.01)	10.1 (±0.02)
D	F	1994/8	485	3,224	1994/8∼1999/8	11.4 (±1.11)	12.0 (±1.22)	10.2 (±0.03)	10.0 (±0.01)
E	F	1995/2	502	3,245	1995/2∼2000/2	12.5 (±0.42)	11.5 (±0.30)	10.5 (±0.05)	10.6 (±0.11)

*Note*. ADSm = adult-directed speech by mother; ADSf = adult-directed speech by father; CDSm = child-directed speech by mother; CDSf = child-directed speech by father; M = male; F = female.

### Procedure

#### Recordings

The database used in this study consists of recordings captured using a DAT recorder (TCD-D10, SONY) and its stereo microphone (ECM-959, SONY) in mono format, with a 16-bit resolution and a sampling rate of 16 kHz. Recording sessions were conducted approximately once a month, whenever the children were in a good mood, to ensure high-quality, naturalistic speech samples. This resulted in > 9,700 recordings for analysis regarding maternal IDS/CDS and > 8,300 recordings for analysis regarding paternal IDS/CDS. The microphone was either handheld by a parent or mounted on a microphone stand. While the majority of recordings took place within the children’s homes, occasional sessions occurred in alternative settings, such as hospitals, the parents’ hometowns, or vacation accommodations. To prioritize capturing naturalistic speech interactions, no specific tasks or structured activities were imposed during the recordings.

Recordings commenced within 1 month of the children’s birth. However, due to familial circumstances, recordings were skipped in certain months, and the duration of recordings varied between months. Detailed information on the recording dates and durations is shown in Table 1 and available through an external source (https://osf.io/n3upf/?view_only=9744b797056648cd80c7ec8f9732e093/?view_only=9744b797056648cd80c7ec8f9732e093).

The recordings are now over two decades old. However, the data were collected using high-fidelity DAT equipment (16-bit, 16 kHz), with clear audio captured in naturalistic home environments. The hardware quality and structured monthly data collection have enabled robust longitudinal analyses. All recordings underwent careful preprocessing to standardize duration and ensure acoustic clarity.

#### Preprocessing of the raw data

Speech data were extracted from the recordings that ranged from approximately 15 min to 1 hr per session. During this extraction process, speech data were segmented based on their context. Temporally adjacent utterances from the same speaker with silent intervals shorter than 500 ms were merged into a single speech file. Conversely, when silent intervals exceeded 500 ms, the speech data were treated as separate events, resulting in two distinct files. This procedure ensured the creation of multiple speech files for each session, accurately reflecting the natural flow of conversation.

To ensure reliable results of frequency analysis, particularly for examining the minimum target frequency of 0.9 Hz in the S-AMPH model used in this study, each speech file required sufficient length. Files shorter than 10 s were merged with temporally adjacent files from the same session to create a file exceeding 10 s in length. To maintain the integrity of individual utterances, 1 s of silence was inserted between the concatenated files. Consequently, all merged data files ranged in length from 10.0 to 12.5 s. Table 1 provides the mean length of the processed speech files. Then, for each speech file longer than 10 s, 3 s of silence were added to both the beginning and end of the speech file to ensure robust analysis across the entire speech waveform. All audio preprocessing steps, including segmentation, silence insertion, and file merging, were performed using MATLAB (Version R2024a). All scripts and processing logs are available upon request.

It is of note that all merged data files ranged in length from 10.0 to 12.5 s in each group (Table 1). This means no large difference in the length of speech data. To systematically verify whether speech file lengths differed significantly among the groups (CDS by mother [CDSm], CDS by father [CDSf], ADS by mother [ADSm], ADS by father [ADSf]), we initially conducted a Shapiro–Wilk test to examine the normality of the data distribution. Given the non-normal distribution revealed by this preliminary analysis, we employed a nonparametric Kruskal–Wallis analysis of variance (ANOVA) with two factors: speech type (CDSm, CDSf, ADSm, ADSf) and participant (A, B, C, D, E). All statistical analyses were performed using jamovi Version 1.2 (The jamovi project, 2021). The results revealed statistically significant differences among the groups (Kruskal–Wallis ANOVA: *H*(3) = 12.56, *p* < 0.05). However, effect sizes, as indicated by eta squared (*η*^2^), were notably small (*η*^2^ ranging from 0.00389 to 0.02974). Such small effect sizes suggest that while statistically significant due to the large number of samples analyzed, these differences are unlikely to reflect meaningful variations in practical terms (Lakens, 2013). To assess practical equivalence of segment durations, we performed ratio-based two one-sided tests on the log scale, concluding equivalence when the 90% confidence interval (CI) of the geometric mean (GM) ratio fell entirely within [0.90, 1.10] (±10%). The ±10% margin was prespecified for interpretability and justified by the observed within-group variability in the present data set (CV ≈ 11–20%; see OSF for the detailed results: https://osf.io/n3upf/?view_only=9744b797056648cd80c7ec8f9732e093), indicating that differences smaller than this range are negligible relative to natural fluctuations. We focused on four prespecified comparisons: ADSf vs. ADSm, CDSf vs. CDSm, ADSf vs. CDSf, and ADSm vs. CDSm. All four prespecified comparisons were equivalent: ADSf vs. ADSm (GM ratio = 0.986, 90% CI [0.966, 1.007]), CDSf vs. CDSm (0.986, [0.984, 0.989]), ADSf vs. CDSf (1.079, [1.063, 1.096]), and ADSm vs. CDSm (1.079, [1.064, 1.095]). Comprehensive details, including exact speech file lengths, are provided in Table 1 and further elaborated in an Excel file on OSF (https://osf.io/n3upf/?view_only=9744b797056648cd80c7ec8f9732e093). For readers seeking more granular detail, the original data can be accessed in Amano et al. (2008; https://doi.org/10.32130/src.INFANT).

### Data Analysis

The S-AMPH model, originally detailed by Leong and Goswami (2015; for further details, see https://www.cne.psychol.cam.ac.uk/wiki-samph/introduction-samph) and used in a series of subsequent studies (Daikoku & Goswami, 2022; Daikoku et al., 2024; Leong et al., 2017; Perez-Navarro et al., 2022), captures hierarchical AM structures in acoustic signals. For detailed methodological steps, readers are encouraged to consult these foundational papers (Daikoku & Goswami, 2022; Leong & Goswami, 2015). Briefly, the analytical process involves these sequential steps:

***Spectral Analysis*:** The raw acoustic signal is passed through a cochlea-inspired an equivalent rectangular bandwidth (ERB_N_)-spaced filter bank (28 channels, covering 100–7,250 Hz). Each spectral channel captures distinct frequency components (e.g., pitch, formants), and their envelopes are extracted using the Hilbert transform. Principal component analysis (PCA) is performed on these envelopes to identify core spectral modulation patterns and, in European languages, reduces dimensionality to five distinct spectral bands.

***Temporal Analysis*:** The raw acoustic signal is next filtered separately into each of the five spectral bands determined from spectral PCA. Each spectral-band-filtered signal undergoes further analysis through a second ERB_N_-spaced filter bank (24 channels, spanning 0.9–40 Hz) to isolate AM rates that, in European languages, correspond to prosodic, syllabic, and onset–rime linguistic components. Temporal PCA is conducted to reveal dominant temporal modulation bands.

**Figure 1.**
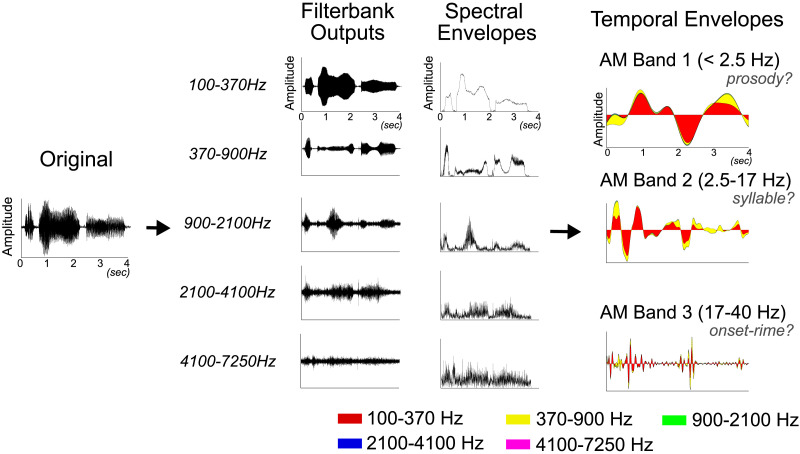
Signal processing workflow in the Spectral-Amplitude Modulation Phase Hierarchy (S-AMPH) model. The figure illustrates the sequential processing steps of the S-AMPH model using a sample waveform derived from this study and illustrating matching linguistic units from prior work in European languages. Step 1 involves passing the raw waveform through an ERB_N_-spaced filter bank, covering an acoustic frequency range of 100–7,250 Hz. This process generates a hierarchical representation of the core spectral (acoustic frequency) and temporal (oscillatory rate) modulation hierarchies within the amplitude envelope of speech, represented as Spectral Envelopes and Temporal Envelopes, respectively. The envelope for each channel is extracted using the Hilbert transform. Dimensionality reduction is achieved via principal component analysis, which identifies patterns of covariation across spectral and temporal channels to determine the number and boundaries of modulation bands. This dimensionality reduction step facilitates the identification of core spectral and temporal modulation bands. This workflow culminates in a cascade of amplitude modulators at varying oscillatory rates, encapsulating the hierarchical structure of speech amplitude modulation (AM). Inspection of the temporal envelopes shows more peaks in the “syllable-rate” AM than in the “prosody-rate” AM. In prior S-AMPH modeling, a strong syllable is perceived when both bands peak together, and a weak syllable is perceived when a trough in the “prosody-rate” AM is aligned with a peak in the “syllable-rate” AM. This figure suggests a more variable alignment in Japanese. In both the spectral and temporal envelope plots, the *x*-axis represents time (in seconds), and the *y*-axis indicates the amplitude values at each timescale. In the temporal envelope plots, the shaded areas represent overlapping temporal envelopes from the five spectral bands (100–370, 370–900, 900–2,100, 2,100–4,100, and 4,100–7,250 Hz). Because these envelopes are highly overlapping and the spectral bands of 100–370 and 370–900 Hz exhibit stronger amplitude power, the yellow and red regions appear most prominently.

Figure 1 visually summarizes this two-stage analytical procedure, indicating the meaning of spectral and temporal channels and clearly labeling all axes and analytical steps. To ensure clarity and reproducibility, all figures were plotted using standardized parameters across speech conditions. The visualization format follows the S-AMPH convention (Leong & Goswami, 2015) but was optimized for the current Japanese corpus to highlight language-specific AM patterns. All processing and plotting scripts are openly available on OSF, allowing readers to reproduce every analytical step.

#### S-AMPH

To exclude the potential effects of sound intensity on spectrotemporal modulation features, the acoustic speech signals were *z*-score normalized (*M* = 0, *SD* = 1). The raw acoustic signals were processed through a 28-channel logarithmically spaced ERB_N_ filter bank, spanning a frequency range of 100–7,250 Hz. The choice of 100 Hz as the lower cutoff frequency follows established methodologies (Moore, 2013), aligning with standard auditory processing thresholds and ensuring robust extraction of spectral modulation features from typical human speech, including male voices. This filter bank emulates the cochlear frequency decomposition observed in a typical human auditory system, enabling the identification of spectral modulation patterns (Dau et al., 1997; Moore, 2013). Technical specifications of the filter bank design are detailed in Stone and Moore (2003), with additional parameter settings and frequency response characteristics provided in Supporting Information Appendix S1, which can be found at https://doi.org/10.1162/NOL.a.226. For each of the 28 frequency channels, signal envelopes were derived using the Hilbert transform, resulting in a set of 28 Hilbert envelopes. The PCA was then applied to these envelopes, uncovering the core spectral modulation patterns by identifying the number and spacing of nonredundant spectral bands based on co-modulation within this high-dimensional ERB_N_ representation. Temporal modulation patterns were subsequently extracted by filtering the raw acoustic signal into the spectral bands identified through the spectral PCA analysis. This two-step process establishes a precise and comprehensive framework for characterizing both spectral and temporal modulation features.

In the spectral PCA, analysis was focused on the top five principal components (PCs) (Figure 1, middle), which collectively explained over 70% (PC1: 46.16%, PC2: 10.45%, PC3: 6.32%, PC4: 4.78%, and PC5: 3.86%) of the total variance in the original acoustic signal. To explore temporal modulation patterns, the raw acoustic signal was filtered into five distinct spectral bands derived from the spectral PCA. Subsequently, the AM envelope within each spectral band was processed through a 24-channel logarithmically spaced ERB_N_ filter bank covering the frequency range of 0.9–40 Hz (see Supporting Information Appendix S2 for further details). The lower cutoff of 0.9 Hz follows the rationale established by Leong and Goswami (2015), who implemented this threshold in the original S-AMPH model to capture prosodic-level AMs while ensuring reliable envelope estimation over naturalistic utterance durations. Lower frequencies (< 0.9 Hz) would necessitate significantly longer continuous speech signals, which are not practical for spontaneous speech recordings, and may also introduce instability in phase calculations.

The Hilbert transform was applied to each of the 24 filtered signals to extract their envelopes. Temporal PCA was then performed to uncover the core temporal modulation structures. Additionally, mean frequency power (MFP) was analyzed to identify which spectral bands made the most substantial contributions to the temporal amplitude structure. For the temporal PCA, only the top three PCs were retained for further analysis, as they cumulatively accounted for over 90% (PC1: 71.38%, PC2: 13.00%, and PC3: 4.68%) of the variance in the original signal (Figure 1, right). Detailed PCA results can be found in Supporting Information Appendix S3. To identify the hierarchical modulation patterns, the absolute values of PC loadings were averaged across all speech samples. Peaks in the grand average PC loading patterns were identified to determine the dominant modulation clusters, while troughs were examined as they delineate the boundaries between co-modulated channel clusters (for detail, see Daikoku & Goswami, 2022). This comprehensive approach highlights the hierarchical organization of spectrotemporal modulation in speech signals.

To identify the number and boundaries of the core modulation bands in both the spectral (acoustic frequencies spanning 100–7,250 Hz) and temporal (oscillatory rates spanning 0.9–40 Hz) domains, the PCA was applied to reduce dimensionality in each domain. PCA, a well-established method for dimensionality reduction in speech research (e.g., Klein et al., 1970; Pols et al., 1973), was employed to uncover the underlying structure of modulation patterns. This analysis focused on the absolute values of component loadings rather than component scores. Component loadings provide insight into the patterns of correlation between high-dimensional channels, reflecting how these channels co-vary. Specifically, PCA loadings were used to identify clusters of co-modulated channels within the spectral (28 channels) and temporal (24 channels) domains, thereby delineating the core modulation bands. These clusters represent the fundamental modulation structures underlying the speech signal. Detailed methodology and additional information on the PCA approach are provided in Supporting Information Appendix S1.

To identify the core spectral and temporal modulation bands, we established criteria grounded in prior research utilizing the S-AMPH framework (Daikoku & Goswami, 2022; Leong & Goswami, 2015). Specifically, to ensure sufficient separation between the inferred modulation bands, a minimum peak-to-peak distance of two channels for spectral PCA and five channels for temporal PCA was imposed. This spacing criterion aimed to prevent overfragmentation of modulation patterns and to reflect meaningful structural organization in speech signals. Additionally, cycles in which the peak-to-peak amplitude was less than 10% of the average peak-to-peak amplitude were excluded, as these were deemed to represent noise rather than meaningful speech cycles. Peaks and troughs were systematically identified across all PCs, with troughs representing the boundaries between modulation bands. These peaks signify the presence of co-modulated clusters, which constitute the primary modulation structures. Boundary edges between bands were delineated based on the most consistent locations of “flanking” troughs, which demarcated the limits of each modulation band. This rigorous approach ensured a robust and interpretable characterization of the spectral and temporal modulation hierarchy in speech.

#### Phase synchronization analyses

We examined multi-timescale phase synchronization between temporal modulation bands by calculating the phase synchronization ratios between adjacent bands identified through the S-AMPH model applied to the speech samples in each language. The PSI was computed for each pair of adjacent AM bands within the S-AMPH representation. Originally developed to quantify synchronization between oscillators of differing frequencies (e.g., muscle activity; Tass et al., 1998), the *n*:*m* PSI has been adapted for analyzing neural oscillatory phase-locking (Schack & Weiss, 2005).

$$PSI=\left|e^{1\left(n\theta 1–m\theta 2\right)}\right|$$ (1)

Here, *n* and *m* denote the relative frequency relationship between the slower and faster AM bands, calculated based on their cycle lengths. For example, if the cycle length of the slowest AM band is 2,000 ms and the AM rhythm nested within this slowest band has a cycle length of 1,500 ms, the resulting *n*:*m* ratio is 4:3. This ratio was determined for each PSI computation. The terms θ1 and θ2 represent the instantaneous phases of the slower and faster AM bands, respectively, at each time point. The expression (*nθ*1 − *mθ*2) calculates the generalized phase difference between the two AM bands, measured as the circular distance (modulus 2*π*) between the instantaneous phase angles. The angled brackets indicate the mean phase difference across all time points, while the absolute value ensures the PSI remains between 0 and 1. A PSI value of 1 corresponds to perfect phase synchronization, indicative of rhythmically regular patterns perceived as a repeating sequence of strong and weak beats. Conversely, a PSI value of 0 denotes a lack of synchronization, perceived as rhythmically irregular or random patterns. This methodology enables the quantification of rhythmic coherence across temporal modulation bands, consistent with prior research (Leong et al., 2017). Then, we averaged the PSI values across all five children within each age point to obtain a single representative value of PSI by age in months, thereby minimizing individual variability and enhancing the stability of the developmental analysis.

Next, we performed the Shapiro–Wilk test for normality on the PSI. Depending on the result of the test for normality, either the parametric or nonparametric (Kruskal–Wallis) one-way ANOVA with a single between-group factor (CDSm, CDSf, ADSm, and ADSf) was applied. Furthermore, to examine developmental changes in phase synchronization, we conducted correlation analyses between child age (in months) and PSI values within each speech condition (CDSm, CDSf). Depending on the result of the test for normality on the PSI, either the Peason’s or Speaman’s correlation test was conducted. To control for multiple comparisons, we applied the Benjamini–Hochberg false discovery rate (FDR) correction procedure. Statistical significance was determined at an FDR-corrected *p*-value threshold of 0.05. Statistical analyses were conducted using jamovi Version 1.2 (The Jamovi Project, 2021). We selected *p* < 0.05 as the threshold for statistical significance.

## RESULTS

### AM Properties

**Figure 2.**
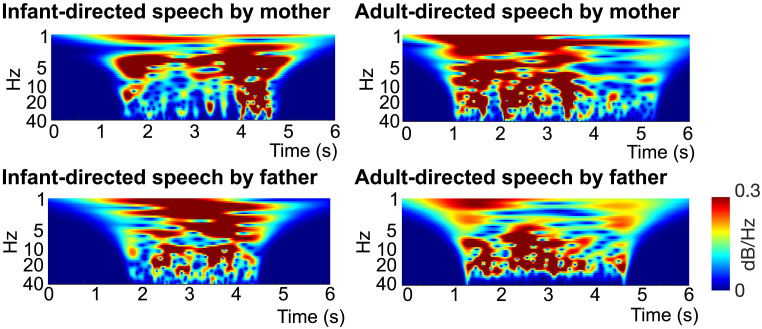
Representative scalograms illustrating amplitude modulation (AM) energy across 0.1–40 Hz in Japanese infant-directed speech (IDS) and adult-directed speech (ADS) from the same speaker. This figure presents scalograms derived from representative examples of speech samples, created using continuous wavelet transform. Each scalogram is based on randomly selected 6-s excerpts of speech and depicts the AM envelopes within these samples. It is important to note that scalograms cannot be directly generated using the Spectral-Amplitude Modulation Phase Hierarchy model due to the application of cochlear filter banks, which result in the loss of distinct boundary frequencies. The *x*-axis represents time (spanning 6 s), while the *y*-axis reflects the modulation rate (ranging from 0.1 to 40 Hz). Amplitude maxima are normalized to 0 dB, and the demodulated outputs are visualized as heat maps, capturing the modulation dynamics across time and frequency domains. IDS displays enhanced low-frequency modulations (∼2–7 Hz) relative to ADS. Color maps and amplitude scales are standardized across panels for direct comparison.

To illustrate the overarching acoustic patterns of the speech analyzed in this study, in Figure 2 we present scalograms displaying the exemplary frequency characteristics. These scalograms were generated using continuous wavelet transforms and are intended to provide a qualitative visualization of the temporal modulation energy across the 0.1–40 Hz range in representative Japanese IDS and ADS utterances.

#### Spectral PCA

The goal of the spectral PCA is to identify the dominant frequency ranges (spectral bands) that comprise the AE of speech in a particular language. A priori, we predicted five spectral bands that would be broadly comparable to those identified for European languages. These bands represent ranges in which energy tends to co-modulate, driven by pitch, formant transitions, and phonetic features. Identifying these bands allows us to decompose the speech signal in a way that aligns with known perceptual and neural processing channels.

**Figure 3.**
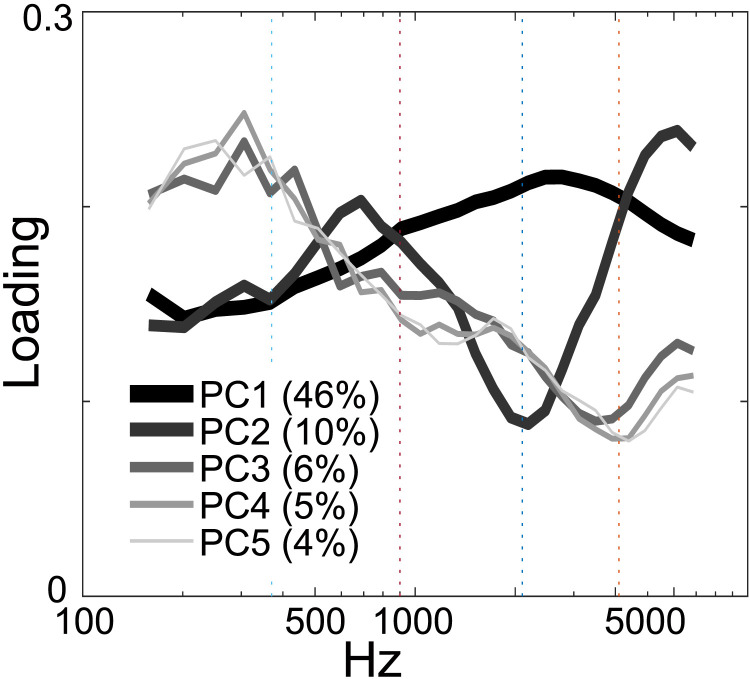
Core spectral modulation rates in speech averaged across families and between infant-directed speech and adult-directed speech. The average absolute value of the spectral principal component (PC) analysis component loading patterns for PC1–PC5 generated by the Spectral-Amplitude Modulation Phase Hierarchy model is depicted. The lines of different thicknesses identify five spectral bands. These align with previous analyses of spectral bands in speech and music that similarly identified five components (e.g., Araújo et al., 2018; Daikoku & Goswami, 2022; Daikoku et al., 2024; Leong & Goswami, 2015; Leong et al., 2017; Perez-Navarro et al., 2022).

The spectral PCA component loading patterns for each of five families are shown in Supporting Information Appendix S2. In each subplot, the lines of different thicknesses indicate different components. The loading patterns for the top five components are similar across the participants. It may be observed that they produced consistent loading patterns, particularly for the first three components (i.e., PC1, PC2, and PC3). Because of the consistency and similarity of the loading patterns across participants, the present study considered the core spectral bands using the grand average PC loading patterns (Figure 3). All of the contribution rates in each component and numbers of PC loading have been deposited to an external source (https://osf.io/n3upf/?view_only=9744b797056648cd80c7ec8f9732e093/).

The first five PCs (PC1–PC5) accounted for an average of 46.16%, 10.45%, 6.32%, 4.78%, and 3.86% of the total variance. The grand averages, loading patterns, and cumulative contributions for each group have been archived in an external repository (https://osf.io/n3upf/?view_only=9744b797056648cd80c7ec8f9732e093/). The spectral PCA analysis uncovered distinct patterns, revealing a hierarchical organization of spectral modulation. PC1 exhibited a prominent peak around 3,000 Hz, reflecting broadly distributed rhythmic energy across temporal channels, as indicated by the absence of distinct peaks and troughs in the PCA loadings. In other words, the modulation energy is relatively uniform, preventing the identification of discrete spectral modulation bands, as suggested by its alignment with average interchannel correlation coefficients (Figure 3). PC2 and PC3 consistently displayed prominent peaks near 300 Hz, corresponding to the range typically associated with fundamental frequency (F0) in speech, particularly in Japanese IDS (Amano et al., 2006; Masataka, 1992; Niwano & Sugai, 2002). The surrounding troughs, such as those around 370 Hz, define the boundaries between co-modulated spectral regions. Additional peaks and troughs in the loading patterns revealed the presence of four more spectral bands (Figure 3 and Materials and Methods), resulting in the establishment of five core spectral bands, each reflecting distinct components of the speech signal. Based on the predefined criteria outlined in the Materials and Methods, the spectral bands were delineated as follows: Band 1 spanning 100–370 Hz, Band 2 encompassing 370–900 Hz, Band 3 covering 900–2,100 Hz, Band 4 ranging from 2,100 to 4,100 Hz, and Band 5 extending from 4,100 to 7,250 Hz. Table 2 provides a detailed summary of the spectral bands and their boundaries.

**Table 2.** Summary of the spectral bands and the flanking boundaries indentified from spectral principal component (PC) analysis of speech.

Spectral bands	Frequency range (Hz)	PC peaks
Band 1	100–370	PC2–PC5
Band 2	370–900	PC2–PC3
Band 3	900–2,100	PC4–PC5
Band 4	2,100–4,100	PC1
Band 5	4,100–7,250	PC2–PC5

Consistent with our methodological decision to retain five PCs, the spectral PCA results identified five spectral bands. This aligns with previous analyses of spectral bands in speech and music that similarly retained five broadly matching components (e.g., Araújo et al., 2018; Daikoku & Goswami, 2022; Daikoku et al., 2024;Leong & Goswami, 2015; Leong et al., 2017; Perez-Navarro et al., 2022). The identification of five distinct spectral bands provides a structural basis for decomposing the speech signal into perceptually meaningful acoustic components. For example, Band 1 (∼100–370 Hz) may capture pitch-related information including F0; Band 2 (∼370–900 Hz) reflects pitch-to-formant transitions; Bands 3–5 may represent higher formant and consonantal energy regions. This decomposition allows for precise modeling of how rhythmic AMs unfold within each spectral band, enabling comparisons of temporal structure across IDS, CDS, and ADS.

#### Temporal PCA

**Figure 4.**
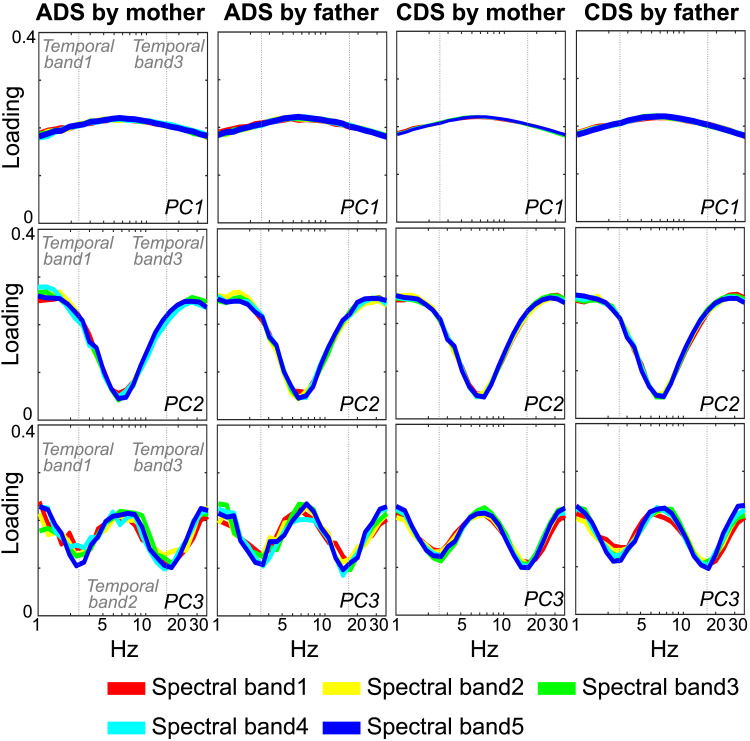
Core temporal modulation rates in speech. The average absolute value of the temporal principal component (PC) analysis component loading patterns for PC1, PC2, and PC3 generated by the Spectral-Amplitude Modulation Phase Hierarchy model is depicted. The model showed an amplitude modulation (AM) hierarchy that consisted of three AM bands. Colors in the figure represent the five spectral bands. PC1 exhibits broad peaks without boundary-defining troughs, while PC2 and PC3 reveal clear segmentation into an AM hierarchy. Together, these components illustrate the three-tiered temporal structure that supports speech encoding. ADS = adult-directed speech; CDS = child-directed speech.

A priori, we expected to find three broad AM bandings, matching the frequencies identified in IDS and CDS in English and Spanish (these were 0.9–2.5 Hz, 2.5–12 Hz, 12–40 Hz; Leong & Goswami, 2015; Leong et al., 2017; Perez-Navarro et al., 2022). Participant-level loading patterns are included in Supporting Information Appendix S2 for validation. Here, we focus on the grand average structure that reflects the three-tiered AM hierarchy (Figure 4). As can be observed, the loading exhibited consistent patterns among the three PCA loading patterns.

Regardless of the types of speech (ADSm, ADSf, CDSm, CDSf), the first to third PCs (PC1–PC3) accounted for 71.38%, 13.00%, and 4.68% in all bands, respectively. Each group’s grand average, loading patterns, and cumulative contribution were deposited to an external source (https://osf.io/n3upf/?view_only=9744b797056648cd80c7ec8f9732e093/).

In all types of speech, PC1 exhibited a moderate peak at 7 Hz across all five spectral bands. This component (Figure 4) likely reflects global coherence across temporal channels, as it lacks the troughs that would mark clear modulation band boundaries. Consequently, the analysis primarily focused on PC2 and PC3. The loading patterns of PC2 revealed two prominent peaks at approximately 1.5 and 30 Hz, consistently separated by distinct flanking troughs around 2.5 and 17 Hz, which were systematically observed across all speech samples (see Supporting Information Figure S2B for cross-participant reproducibility). Furthermore, the maxima at ∼1.5, ∼7, and ∼30 Hz fall within the modulation ranges associated with prosodic-, syllabic-, and phonetic-rate processes (Giraud & Poeppel 2012; Gross et al., 2013; Leong & Goswami 2015). Following Leong and Goswami (2015), these spectral peaks can be interpreted as reflecting dominant temporal regularities that organize the rhythmic hierarchy of speech, whereas troughs indicate the relative absence of such regularity. These frequencies correspond to delta, theta, and beta/low-gamma cortical oscillations known to track the speech AE, supporting the interpretation that the observed maxima represent genuine modulation peaks rather than baseline plateaus. PC3 loading patterns exhibited distinct peaks at approximately 1.5, 7, and 30 Hz. Flanking troughs for these peaks were identified at approximately 2.5 and 17 Hz. Based on the predefined criteria (see Materials and Methods), the temporal PCA results supported the existence of three core temporal AM bands, delineated by two boundaries (Table 3). These findings align well with previous research on IDS, CDS, and music, supporting the cross-domain and cross-linguistic relevance of these temporal modulation bands (Araújo et al., 2018; Daikoku & Goswami, 2022; Daikoku et al., 2024; Leong & Goswami, 2015; Leong et al., 2017; Perez-Navarro et al., 2022). However, the second-slowest band (Band 2) is notably wider in Japanese compared to prior CDS and IDS analyses of European languages, where the boundary has been ∼12 Hz.

**Table 3.** Summary of the three temporal bands and the two flanking boundaries indentified from temporal principal component (PC) analysis.

Temporal bands	Frequency range (Hz)	PC peaks
Band 1	0.9–2.5	PC2, PC3 in spectral bands 1–5
Band 2	2.5–17	PC1, PC3 in spectral bands 1–5
Band 3	17–40	PC2, PC3 in spectral bands 1–5

### Multi-Timescale Phase Synchronization

To investigate the extent of phase synchronization between modulation bands, we generated 5 × 3 temporal modulation envelopes corresponding to the five spectral bands and three temporal modulation bands. Notably, the MFP was significantly higher in spectral Band 1 (the pitch band of the human voice) and Band 2 (the pitch-to-formant transition band) compared to the other bands (Supporting Information Appendix S3, Table a), while the overall trend showed a gradual increase in MFP from Band 5 to Band 1 (repeated-measures ANOVA, all *p* < 0.01; Tukey’s post hoc test). Detailed statistical results have been deposited in an external repository (https://osf.io/n3upf/). This heightened power would be expected given the 1/*f* nature of the speech power spectrum, in which lower-frequency bands naturally exhibit higher energy in all languages.

We then examined the PSIs (Leong et al., 2017) between the two slowest AM bands. Based on previous analyses of both speech and various musical genres (Daikoku & Goswami, 2022; Leong et al., 2017; Perez-Navarro et al., 2022), we predicted a priori that the integer ratio of 1:2 would emerge as the dominant ratio, signaling the presence of a metrical beat structure. In Western music (not speech), there are also dominant 1:3 and 2:3 beat structures. The PSI analyses here demonstrated a high degree of consistency across participants (see Supporting Information Appendix S4). However, in contrast to European languages, the 1:2 and 1:3 integer ratios both exhibited equally high PSIs within the “prosodic-syllabic” AM bands. Additionally, the 2:3 integer ratios in the S-AMPH modeling showed relatively high PSIs compared to other integer ratios. Accordingly, the equal dominance of 1:2 and 1:3 ratios in the current analysis appears to be unique to Japanese. The prior studies of European languages all reported that the PSI of the 1:2 ratio was significantly stronger in IDS/CDS than in ADS (Daikoku & Goswami, 2022; Leong et al., 2017; Perez-Navarro et al., 2022). While our study revealed similar differences in PSI when comparing the 1:2 ratio in Japanese IDS/CDS with Japanese ADS, with significantly stronger rhythmic coupling in IDS/CDS compared to ADS (*p* < 0.05; Figures 4B and 4C), we found similar differences in PSIs between ADS and IDS/CDS for the 1:3 ratio (*p* < 0.05, FDR corrected) and for the 2:3 ratio (*p* < 0.05, FDR corrected). Accordingly, statistical comparisons revealed significant differences in PSI values between IDS/CDS and ADS conditions for all integer ratios examined.

Specifically, for the ***1:2 ratio***, PSI values were significantly higher in CDS (father: *M* = 0.112, *SE* = 0.00107; mother: *M* = 0.112, *SE* = 0.00136) compared to ADS (father: *M* = 0.0981, *SE* = 0.00313; mother: *M* = 0.105, *SE* = 0.00283) (CDSf vs. ADSf: *t*(192) = −4.882, FDR-corrected *p* < 0.001, Cohen’s *d* = 0.996, 95% CI [−1.411, −0.581]; CDSm vs. ADSm: *t*(192) = −2.341, FDR-corrected *p* = 0.03, Cohen’s *d* = 0.481, 95% CI [−8.9, −0.073]). Similar patterns were observed for the ***1:3 ratio*** (CDS father: *M* = 0.118, *SE* = 0.000653; CDS mother: *M* = 0.113, *SE* = 0.000882 vs. ADS father: *M* = 0.109, *SE* = 0.002; ADS mother: *M* = 0.106, *SE* = 0.0025; *t*(97) = 3.67, FDR-corrected *p* = 0.008) (CDSf vs. ADSf: *W* = 5.76, FDR-corrected *p* < 0.001; CDSm vs. ADSm: *W* = 4.17, FDR-corrected *p* < 0.017) and the ***2:3 ratio*** (CDS father: *M* = 0.11, *SE* = 0.000871; CDS mother: *M* = 0.109, *SE* = 0.000961 vs. ADS father: *M* = 0.0982, *SE* = 0.00237; ADS mother: *M* = 0.1, *SE* = 0.00302; *t*(97) = 3.23, FDR-corrected *p* = 0.015) (CDSf vs. ADSf: *W* = 7.173, FDR-corrected *p* < 0.001; CDSm vs. ADSm: *W* = 6.062, FDR-corrected *p* < 0.001). This pattern suggests that, in addition to the typically dominant 1:2 synchronization found in European languages, indicative of a temporally regular alternation between strong and weak syllables, Japanese IDS/CDS exhibits other and equally strong AM-phase relationships. These additional ratios may reflect language-specific adaptations to moraic rhythm structure in Japanese, allowing for the simultaneous encoding of both syllabic and subsyllabic (moraic) timing units.

**Figure 5.**
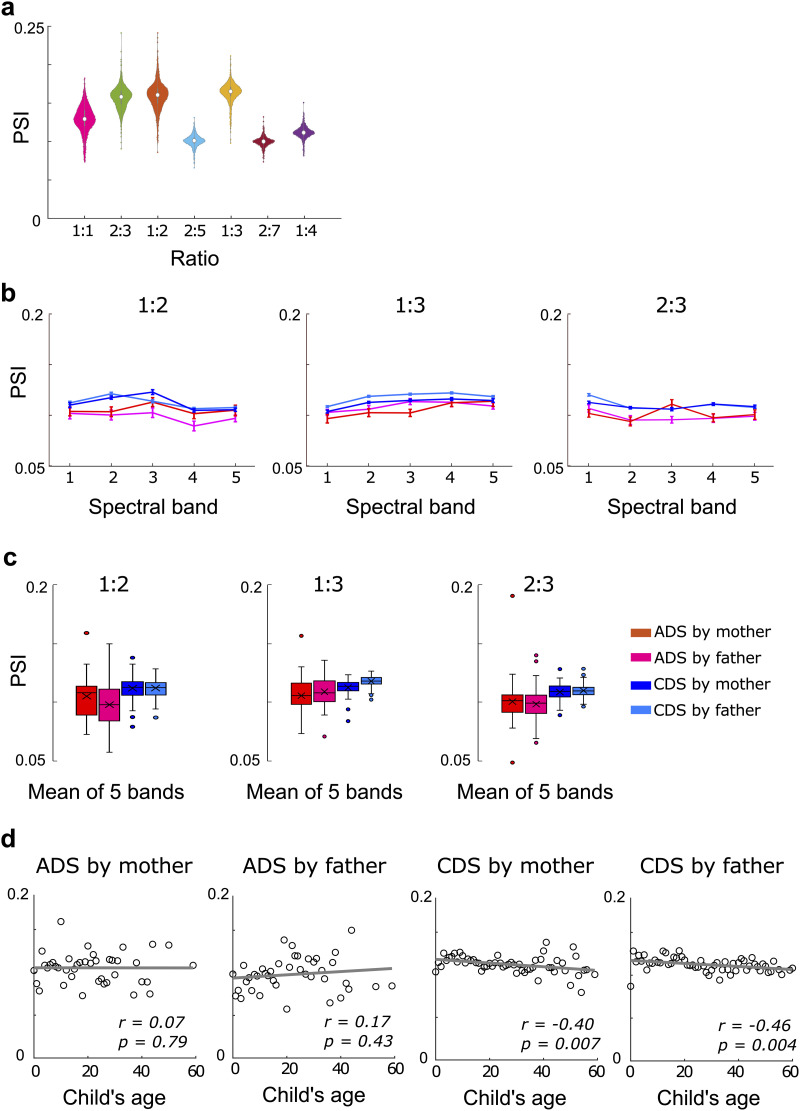
Phase synchronization index (PSI) for each type of speech. Each integer ratio (A) in PSI averaged across the five spectral bands. The simpler integer 1:2 and 1:3 ratios are as prevalent as each other in this corpus, and the 2:3 ratio is also prominent. The 2:3 ratio may, however, reflect a mathematical averaging effect regarding 1:2 and 1:3. The PSI in the 1:2 and 1:3 ratios was significantly stronger in child-directed speech (CDS) compared to adult-directed speech (ADS) (B and C). Additionally, within CDS, a decline in the PSI was observed as the child’s age increased (D).

Of further note, within IDS/CDS, a decline in the PSI was observed as the child’s age increased (see Figure 5D) (CDSf: Spearman’s rho = −0.458, *df* = 56, *p* = 0.00375, FDR corrected; CDSm: Spearman’s rho = −0.4, *df* = 54, *p* = 0.0067, FDR corrected). This suggests developmental changes in AM structure over time and was not previously tested for European languages as all prior data sets were cross-sectional. Although the regression lines in Figure 5D may appear visually flat, this is due to the compressed range of PSI values on the *y*-axis. The statistical significance of the correlation reflects a consistent monotonic trend across time points with relatively low variability, rather than large absolute changes in PSI. Complete statistical details, including all descriptive and inferential statistics, are provided in Supporting Information Appendix S3 and accessible online via OSF (https://osf.io/n3upf/?view_only=9744b797056648cd80c7ec8f9732e093).

## DISCUSSION

Here, we applied a language-blind computational model of linguistic rhythm based on features of the AE to compute the physical acoustic stimulus characteristics that characterize Japanese IDS, CDS and ADS. The S-AMPH model identified systematic temporal modulation patterns and PSIs across these three speech types, which largely matched prior findings in European syllable-timed (French, Spanish, Portuguese) and stress-timed (English, German) languages (Araújo et al., 2018; Daikoku & Goswami, 2022; Daikoku et al., 2024; Leong & Goswami, 2015; Perez-Navarro et al., 2022). As in these European languages, the modeling demonstrated five spectral bandings and three temporal bandings, with very similar ranges in Hz irrespective of whether the Japanese mother or the Japanese father was the speaker. This is important because an extensive neurophysiological literature demonstrates that the temporal modulation patterns observed here align with the frequency bands (delta, theta, beta/gamma) of the cortical oscillatory networks engaged during speech processing (Giraud & Poeppel, 2012; Gross et al., 2013). Neural studies have reported consistently that there is cortical tracking of envelope (AM) information matching the delta (∼0.5–4 Hz) and theta (∼4–8 Hz) frequencies as well as cortical tracking of envelopes at higher frequencies in the beta and gamma bands (e.g., Keitel et al., 2025, for a review; Poeppel & Assaneo, 2020). Thus, it is plausible that the universal temporal modulation structures identified acoustically by the current modeling would facilitate cortical speech tracking across diverse language environments and across languages with different linguistic rhythm types. As also found in European languages, the PSIs between the two slowest AM bandings were significantly higher in speech directed to infants and children than in speech directed to adults. This suggests a language-teaching function regarding these AM bandings in IDS and their PSIs. This is supportive of the production of universal acoustic physical structures by speakers of IDS and CDS, which may serve as a foundational perceptual scaffold for infant language development.

However, the modeling also demonstrated some important differences between IDS and CDS in Japanese and previously modeled European languages. One notable cross-language difference was that the second slowest AM banding in Japanese IDS/CDS was elongated compared to European languages, spanning 2.5–17 Hz instead of 2.5–12 Hz. This could potentially reflect an acoustic adaptation linked to moraic rhythm. As noted previously, moraic units are thought to occur at ∼8–10 Hz (Han, 1994; Peter et al., 2022; Warner & Arai, 2001). A wider AM band as identified in our acoustic analyses could facilitate moraic parsing by encompassing the rhythmic range associated with both syllabic and moraic units. This broader band could plausibly accommodate neural tracking of moraic structures by allowing flexibility in capturing subsyllabic units that may be centered on ∼8/10 Hz alongside slower units centered on ∼4/5 Hz. Thus, a broader “syllabic-rate” band in Japanese IDS and CDS may support the development of moraic parsing. This broader band may enable simultaneous parsing of syllabic and subsyllabic (moraic) units, consistent with findings by Sato et al. (2010, 2012), who demonstrated that moraic sensitivity in Japanese infants was still emerging at 10 months of age. Alternatively, there may be other spectrotemporal fluctuations in IDS that enable beat perception and synchronized neural activity, as shown recently for music (Weineck et al., 2022). These features, also called spectral flux, may be complementary to the AM-driven hierarchy of temporal information documented here and may possibly contribute to the perception of moraic timing.

A second important cross-language difference was that 1:3 ratios were as frequent as 1:2 ratios when the PSIs were computed for the two slowest AM bandings. Rather than the perception of rhythmic “beat” being driven only by a metronome-like 1:2 ratio, it was equally likely to be driven by a 1:3 ratio. A PSI of 1:2 indicates a clear metrical beat with two faster cycles nested within one slower cycle, such as two syllables per prosodic foot. For example, in the phrase “JACK and JILL went UP the HILL,” stressed syllables are perceived when peaks in the slower delta-band rhythm and peaks in the faster theta-band rhythm are aligned (“JACK”), and unstressed syllables are perceived when a trough in the delta-band rhythm aligns with a peak in the faster theta-band rhythm (“and”). A PSI of 1:3 means that there are also triplet beats (an English example would be “PUSS-y-cat PUSS-y-cat, WHERE have you BEEN?” with three syllables per foot). Presumably a PSI of 1:3 could accommodate moraic syllabic parsing such as in the example PA-N for the first syllable of PANDA, where there are two rhythmic units in the stressed syllable PAN and one in the unstressed syllable DA (hence a PSI of 1:3). One possible conclusion is that Japanese-learning infants are extracting both kinds of rhythmic structure from IDS simultaneously. When modeling Western music (jazz, classical, rock; Daikoku & Goswami, 2022), 1:3 ratios were also prominent, supporting the importance of AM-ratio structures in contributing to the perception of rhythmic patterning. However, given that a second significant modulation peak at ∼10 Hz in this AM band was absent, it is also possible that other spectrotemporal fluctuations may contribute more strongly to the extraction of morae.

These integer ratios (1:2, 1:3) derived from the PSI are critical to the perception of speech rhythm, as they represent rhythmic synchronization between slower and faster AM bands. The claim here is that these ratios reflect metrical regularities that may help infants segment speech and learn rhythmic patterns. In contrast to prior studies of European languages where a 1:2 ratio predominates (Leong & Goswami, 2015; Leong et al., 2017; Perez-Navarro et al., 2022), our PSI analysis revealed equally dominant 1:2 and 1:3 ratios. This pattern may support simultaneous encoding of temporally different rhythmic units and suggests that the acoustic temporal structure of Japanese IDS supports both syllabic and moraic rhythm acquisition.

It should also be noted that our findings complement and extend those reported by Varnet et al. (2017), who analyzed ADS modulation spectra across multiple languages, including Japanese. Their results demonstrated distinct spectral and temporal modulation characteristics in Japanese relative to other languages, but without identifying a matching AM phase hierarchy. Specifically, they reported that the maximum modulation index reached at the peak was significantly lower in Japanese ADS than in English ADS, reflecting the mora-timed nature of Japanese. Nevertheless, they concluded that the human auditory system is optimized for the processing of temporal modulation patterns in all languages. Our S-AMPH analysis similarly highlights language-specific temporal modulation characteristics, notably the broader “syllabic-rate” AM band in Japanese speech, which potentially supports moraic parsing.

The assumption that Japanese-learning infants can parse both syllabic and moraic units from the speech stream early in life is supported by prior work showing that Japanese infants’ use of moraic units is weaker than English- and French-learning infants’ use of syllable and stress information (Sato et al., 2010, 2012). Sato and her colleagues have demonstrated that Japanese-learning infants do not discriminate monomoraic from bimoraic syllables until 10 months of age (Sato et al., 2010, 2012), suggesting that sensitivity to moraic structure develops gradually. Our findings imply that the acoustic input of Japanese IDS and CDS contains the necessary structures to support this later-developing perceptual ability. Our findings further align with the classical linguistic literature that emphasizes moraic rhythm as a fundamental perceptual and acoustic feature uniquely characterizing Japanese. Anne Cutler and colleagues demonstrated extensively that moraic units significantly influence speech perception and language processing among adult native Japanese speakers, who preferentially segment speech based on moraic boundaries (Cutler & Otake, 1994; Otake et al., 1993). Our analysis highlights how AM structures in Japanese IDS/CDS potentially support the *development* of moraic parsing and processing from infancy.

Furthermore, and as also found in previous studies of IDS, CDS and ADS in European languages, notable differences in the strength of PSIs were observed between Japanese IDS/CDS and Japanese ADS. Japanese IDS/CDS exhibited significantly stronger phase synchronization than Japanese ADS for the two slowest modulation bands. This elevated PSI contributes to the exaggerated prosodic features of IDS/CDS, providing the learning brain with more salient rhythmic acoustic cues (Amano et al., 2006; Leong et al., 2017; Perez-Navarro et al., 2022). Within Japanese IDS/CDS, we also observed a decline in PSI as children aged. The observed decline in PSI values with child age parallels findings from Amano et al. (2006), indicating that multiple rhythmic and prosodic cues in IDS are dynamically adapted by parents as language acquisition progresses. This is the first longitudinal data set modeled using the S-AMPH, and this developmental trend in PSI is not unexpected. It suggests that as children become more capable of parsing speech and producing language and as their growing comprehension brings top-down processes into play, the prosodic emphasis in caregiver speech diminishes, moving gradually toward the acoustic physical structures that characterize ADS. Importantly, however, the data analyzed here were recordings of natural conversational speech. In deliberately rhythmic speech, such as poetry and proverbs, ADS continues to exhibit significantly stronger “prosodic–syllabic” PSIs than conversational speech (Araújo et al., 2018).

The longitudinal corpus used in this study has also been utilized in earlier investigations of Japanese IDS, thereby demonstrating that all the different language-teaching characteristics of IDS are present at the same time (different AM structure to ADS, heightened pitch, exaggerated pitch range, and hyperarticulation of vowels; Amano et al., 2006). Amano et al. (2006) conducted a detailed analysis of the F0 of IDS, reporting that F0 decreases nearly linearly with infant age. Amano et al. also found that in IDS, the F0 is significantly higher than in ADS during the period from birth to around 18 months, the age at which infants typically begin producing two-word utterances. Over this period, F0 in Japanese IDS decreases as the child ages. Our findings provide complementary evidence of an accompanying decline in the phase synchronization between the two slowest AM bandings. Via our novel focus on AM hierarchies, we demonstrate how the different language-teaching characteristics of IDS change over a similar time frame. The data are consistent with the notion that Japanese IDS prosody is tailored to the language’s phonological system (Mazuka et al., 2015), yet it also supports the idea that IDS exhibits common acoustic patterns across languages (Goswami, 2022). Our results thus extend the IDS studies by Mazuka and her colleagues by revealing that the temporal modulation structure (AM bands) of Japanese IDS aligns with patterns observed in other languages’ IDS, suggesting a potentially universal aspect of how IDS is structured to engage infant auditory processing.

One limitation of the current study is the small sample size of six speakers, dictated by the logistical challenges of obtaining long-term naturalistic recordings over 5 years. Although longitudinal studies provide unparalleled insights into individual developmental trajectories, the small number of speakers may limit the generalizability of the findings. Nevertheless, the first study to apply the S-AMPH model to English CDS also used six speakers (Leong & Goswami, 2015), who each recited 44 nursery rhymes yielding 264 speech samples for analysis. This first English study identified temporal and spectral bandings, which were replicated in a later study of IDS with 24 speakers (Leong et al., 2017). The Spanish CDS study had 18 speakers (Perez-Navarro et al., 2022). Future research could aim to replicate the current study using larger data sets. Additionally, while the AM hierarchy and PSI analyses revealed robust patterns, further studies could examine cross-linguistic comparisons involving other mora-timed languages, such as Gilbertese or Slovak, to better understand whether the rhythmic characteristics observed here are unique to Japanese or reflect broader linguistic typologies.

In conclusion, this study contributes to the growing body of research exploring the rhythmic properties of speech from an AE-perspective with special reference to IDS/CDS. By uncovering broadly similar AM hierarchies and PSI patterns in Japanese speech as found in European stress-timed and syllable-timed languages, we provide evidence for the universality of rhythmic structures in human communication. At the same time, the data highlight the temporal dynamics unique to Japanese IDS/CDS, which could accommodate moraic timing. Our modeling deepens understanding of how universal physical acoustic structures may enable cortical tracking across languages while simultaneously accommodating linguistic differences in rhythm type, offering unique insights into the fundamental mechanisms of language acquisition.

## Funding Information

Tatsuya Daikoku, Japan Society for the Promotion of Science (https://dx.doi.org/10.13039/501100001691), Award ID: 25H00448. Tatsuya Daikoku, National Institute for Japanese Language and Linguistics (https://dx.doi.org/10.13039/100018205). Usha Goswami, Kadas Prize Foundation (https://dx.doi.org/10.13039/100014648). The funders had no role in study design, data collection and analysis, decision to publish, or preparation of the manuscript.

## Author Contributions

**T.D.**: Conceptualization; Data curation; Formal analysis; Investigation; Methodology; Visualization; Writing – original draft; Writing – review & editing. **U.G.**: Conceptualization; Methodology; Writing – review & editing.

## Code and Data Availability Statements

In this paper, we used “NTT Infant Speech Database (INFANT)” provided by Speech Resources Consortium, National Institute of Informatics (https://doi.org/10.32130/src.INFANT). All of anonymized data files analyzed have been deposited to an external source (https://osf.io/n3upf/). The other data and results of statistical analysis are shown in the Supporting Information.

## Supplementary Material


