# Supplementary material for: The Amplitude Modulation Structure of Japanese Infant- and Child-Directed Speech: Longitudinal Data Reveal Universal Acoustic Physical Structures That Accommodate Both Syllabic and Moraic Timing
Source: Neurobiol Lang (Camb). 2026 Mar 26;7:NOL.a.226. doi: 10.1162/NOL.a.226 (PMC13065094; doi:10.1162/NOL.a.226)
Supplement: Supplementary file 1 [file nol-07-226-s001.pdf]

** This supplementary material is reprinted from Daikoku, Lee, Goswami, 2024, Royal Society Open Science*

Fig. a. Signal Processing Steps in S-AMPH Model.

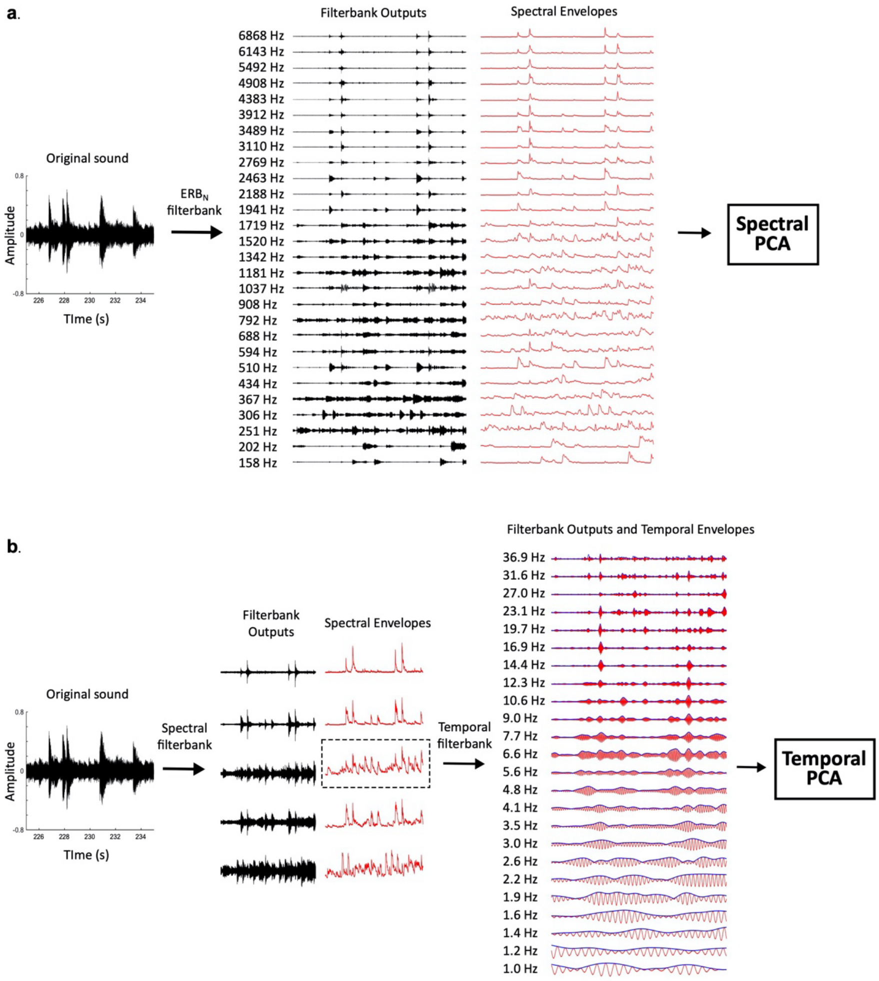

(**a**) Original sound is passed through an ERB$_N$-spaced filterbank, yielding a set of high-dimensional spectral channel outputs (reprinted from Daikoku, Lee, Goswami, 2024, Royal Society Open Science). The envelope is extracted from each spectral channel output using the Hilbert transform, and these envelopes are entered into the spectral PCA to identify patterns of covariation across spectral channels. (**b**) The original sound is passed through a low-dimensional spectral filterbank, yielding a small set of core spectral band outputs. The parameters of the low-dimensional spectral filterbank were determined in the Spectral PCA procedure (**a**). The envelopes are extracted from each spectral band output using the Hilbert transform. Each envelope is further passed through a high-dimensional modulation filterbank, yielding a set of high-dimensional modulation rate envelopes. This rate-filtering is performed for each spectral band envelope, but for simplicity, only the modulation rate envelopes from a single spectral band are shown in this figure. Finally, the power profiles of the modulation rate envelopes (bold blue line) are entered into a temporal PCA to identify patterns of covariation across modulation rates.

Table. a. ERB filterbank (for 28 filter bank) and the frequency response characteristics.

Edge Number	Edge (Hz)
1 (low-pass)	100
2	137
3	179
4	225
5	277
6	334
7	398
8	470
9	549
10	638
11	736
12	846
13	969
14	1105
15	1257
16	1426
17	1614
18	1824
19	2057
20	2317
21	2607
22	2930
23	3289
24	3689
25	4135
26	4631
27	5184
28	5800
29	6486
30	7250

Fig. b. frequency response characteristics (28 filterbanks).

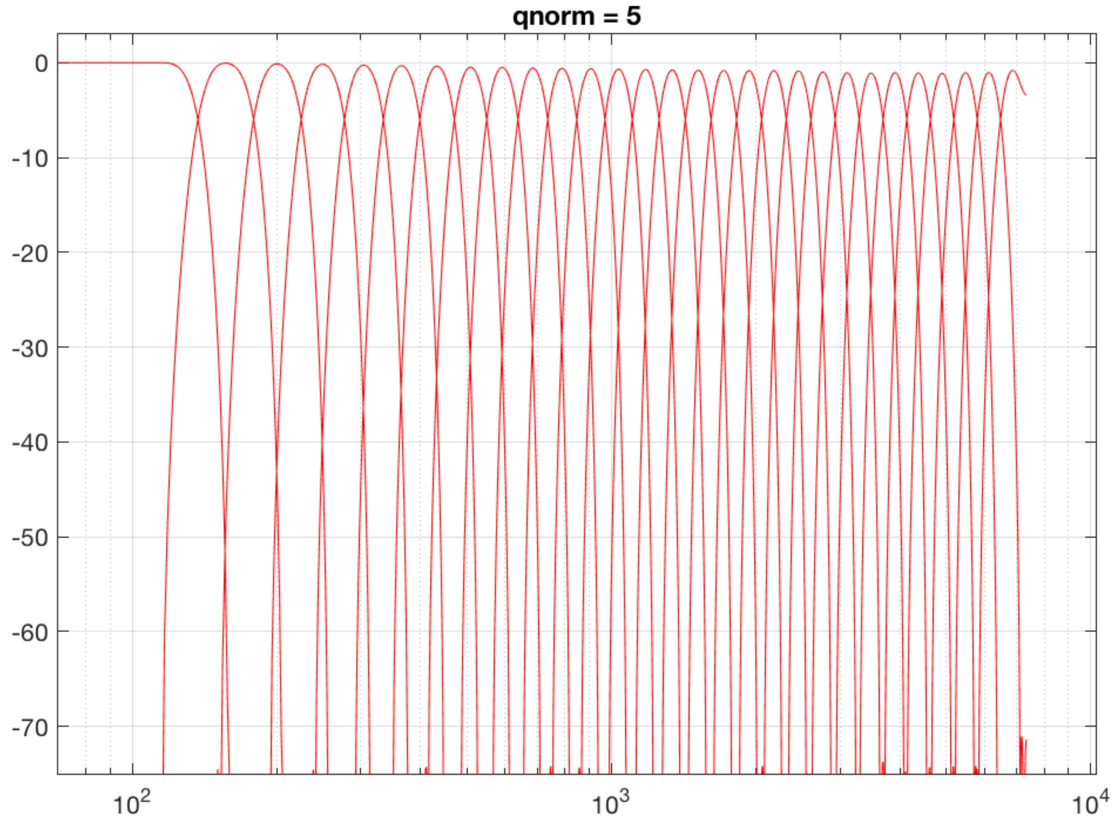

Modulation filterbank (for 24 filter bank) and the frequency response characteristics (Fig. d and e).

Edge Number	Edge (Hz)
1 (low-pass dummy	0.79
2	0.93
3	1.09
4	1.27
5	1.49
6	1.74
7	2.03
8	2.38
9	2.78
10	3.25
11	3.80
12	4.45
13	5.20
14	6.08
15	7.11
16	8.32
17	9.72
18	11.38
19	13.30
20	15.56
21	18.20
22	21.28
23	24.89
24	29.11
25	34.04
26	39.81

Fig. c. frequency response characteristics (5 filterbanks).

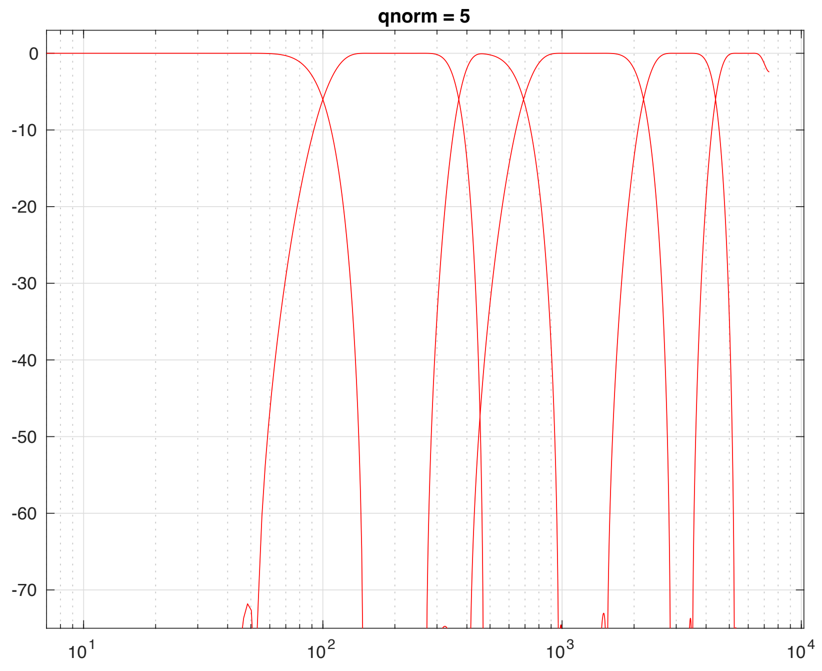

Fig. d. frequency response characteristics (24 filterbanks).

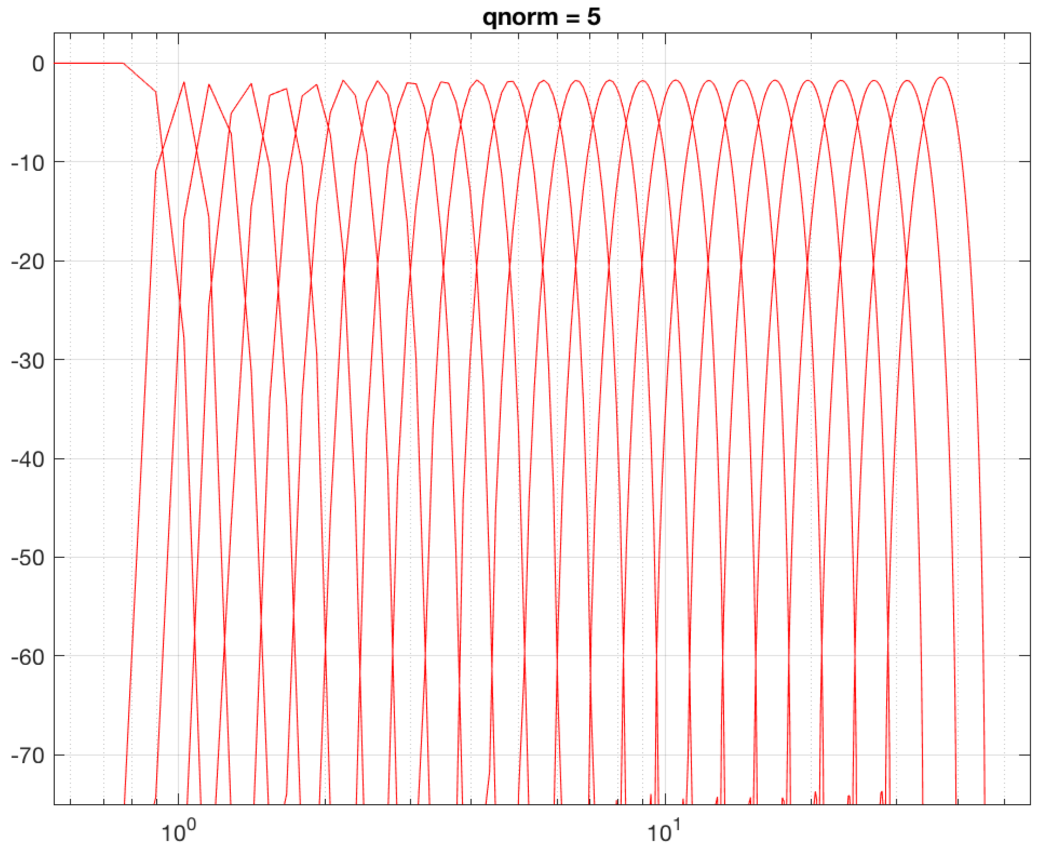

Figure a: Individual Variation in Spectral PCA Component Loadings and the cumulative accounts

Grand average of adult speech

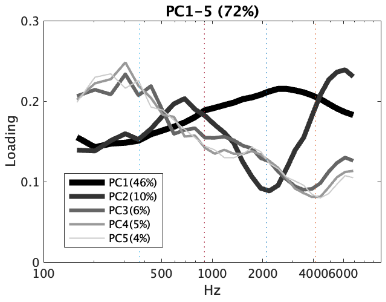

- ADS_father = adult-directed speech by father
- ADS_mother = adult-directed speech by mother
- CDS_father = child-directed speech by father
- CDS_mother =child-directed speech by mother

Grand average of the five families

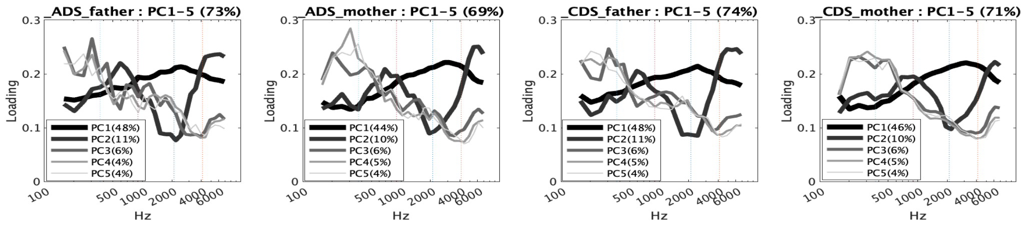

Each of five families (family ID = sa, kk, sk, ma, mk)

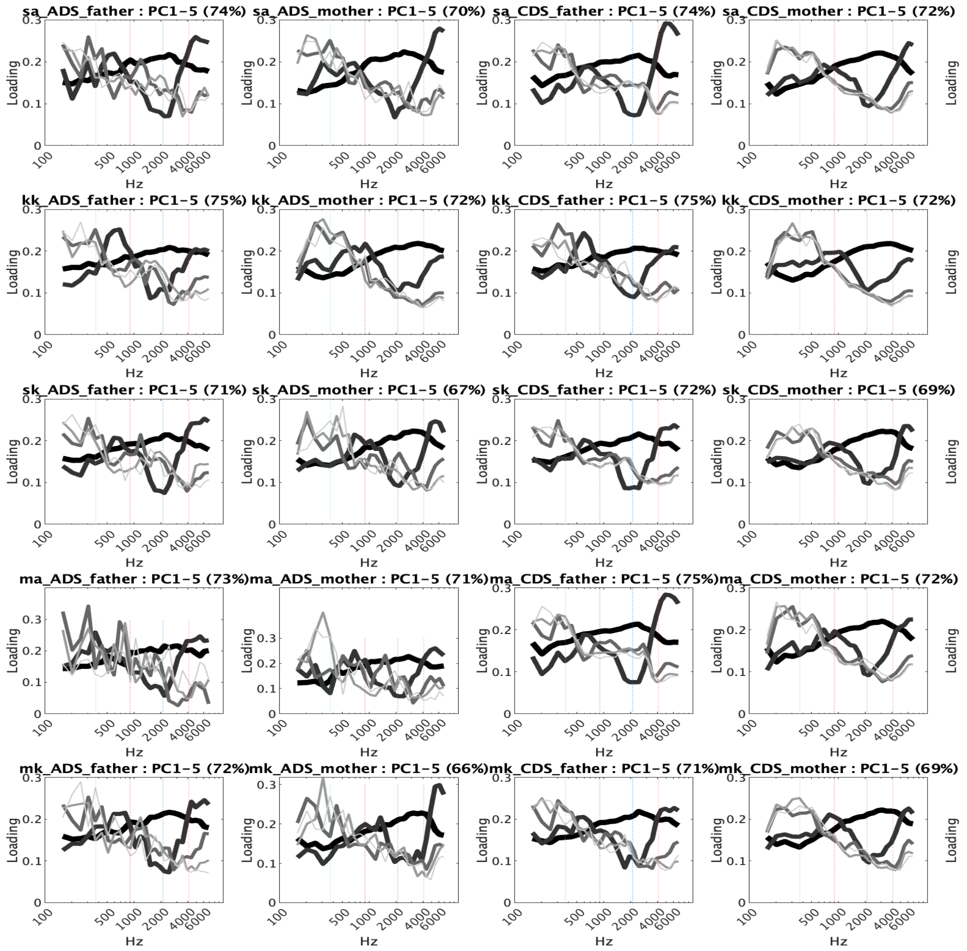

Figure a shows the spectral PCA component loading patterns for each of five families (family ID = sa, kk, sk, ma, mk). In each subplot, the lines of different thicknesses indicate different components. More important (lower-numbered) components with larger contribution rates are shown in a thicker line. The loading patterns for the top 5 components are similar across the participants. It may be observed that they produced consistent loading patterns, particularly for the first 3 components (i.e., PC1, PC2, and PC3). We, however, also find the individual variation at each spectral modulation band. Because of the consistency and similarity of the loading patterns across participants, the present study considered the core spectral bands using the grand average PC loading patterns for each type of speech and for each of five family. All of the contribution rates in each component and numbers of PC loading have been deposited to an external source (https://osf.io/n3upf/).

Table a. Summary of the spectral bands and the flanking boundaries indentified from spectral PCA in each of adult speech.

Spectral bands	Frequency range (Hz)	PC Peaks
Band 1	100-370	PC2-PC5
Band 2	370-900	PC2-PC3
Band 3	900-2100	PC4-PC5
Band 4	2100-4100	PC1
Band 5	4100-7250	PC2-PC5

Figure b: Individual Variation in Temporal PCA Component Loadings

Grand average of adult speech

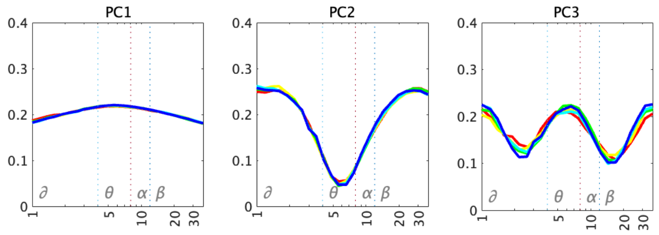

- ADS_father = adult-directed speech by father
- ADS_mother = adult-directed speech by mother
- CDS_father = child-directed speech by father
- CDS_mother =child-directed speech by mother

Grand average of the five families

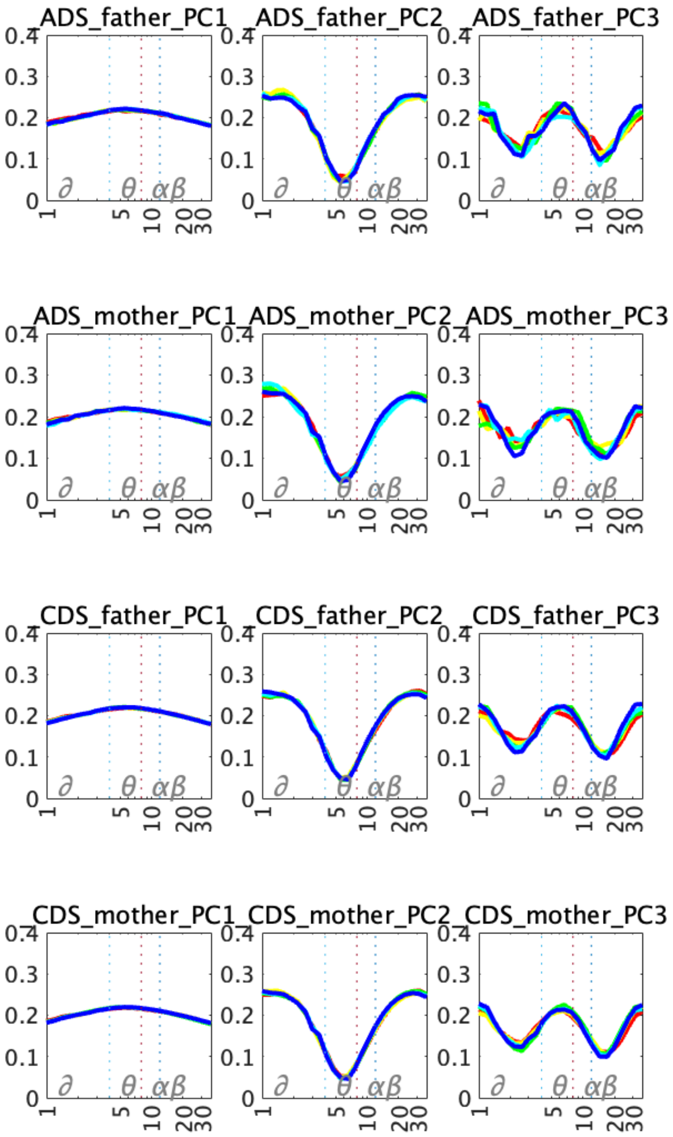

Each of five families (family ID = sa, kk, sk, ma, mk)

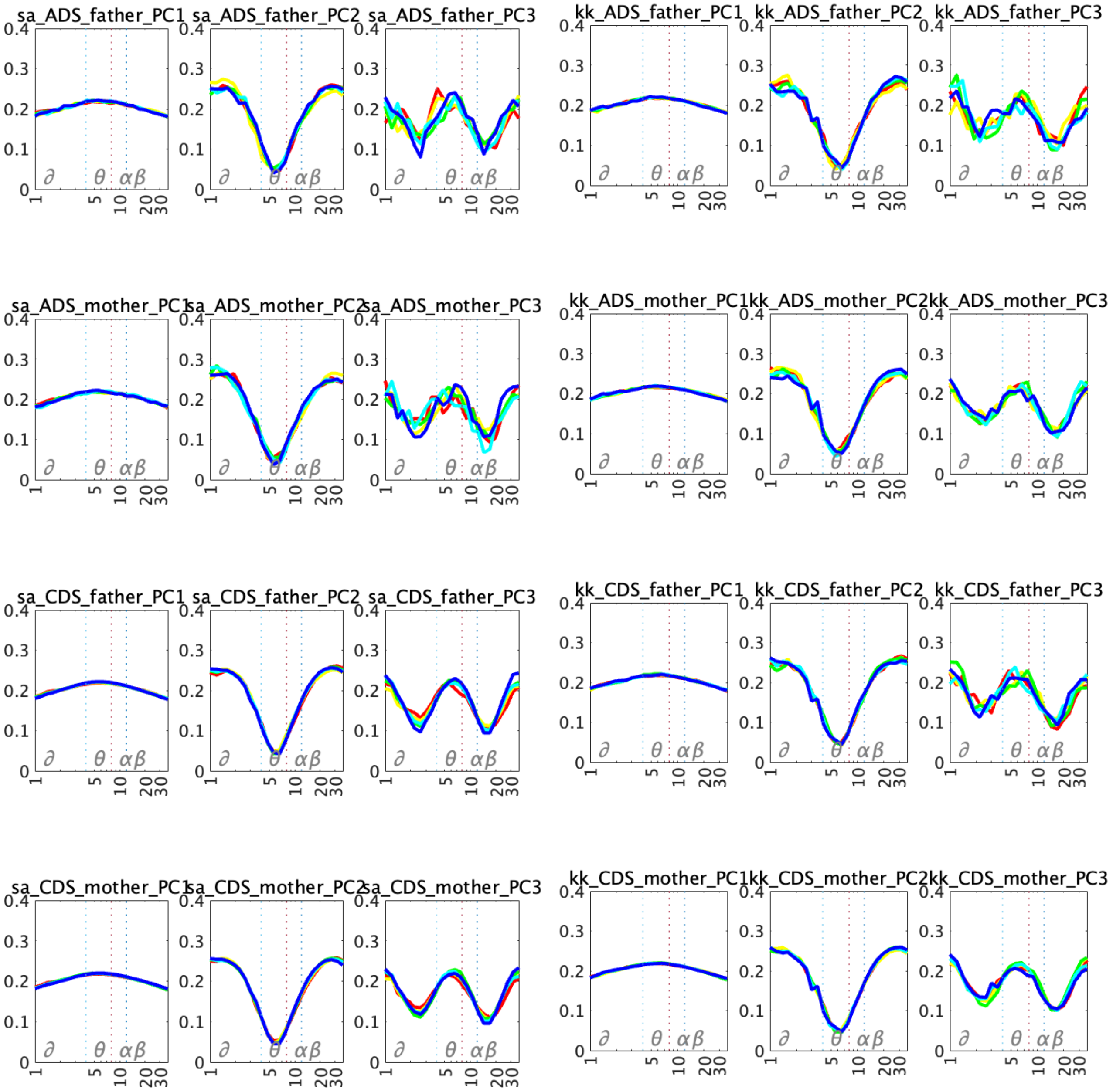

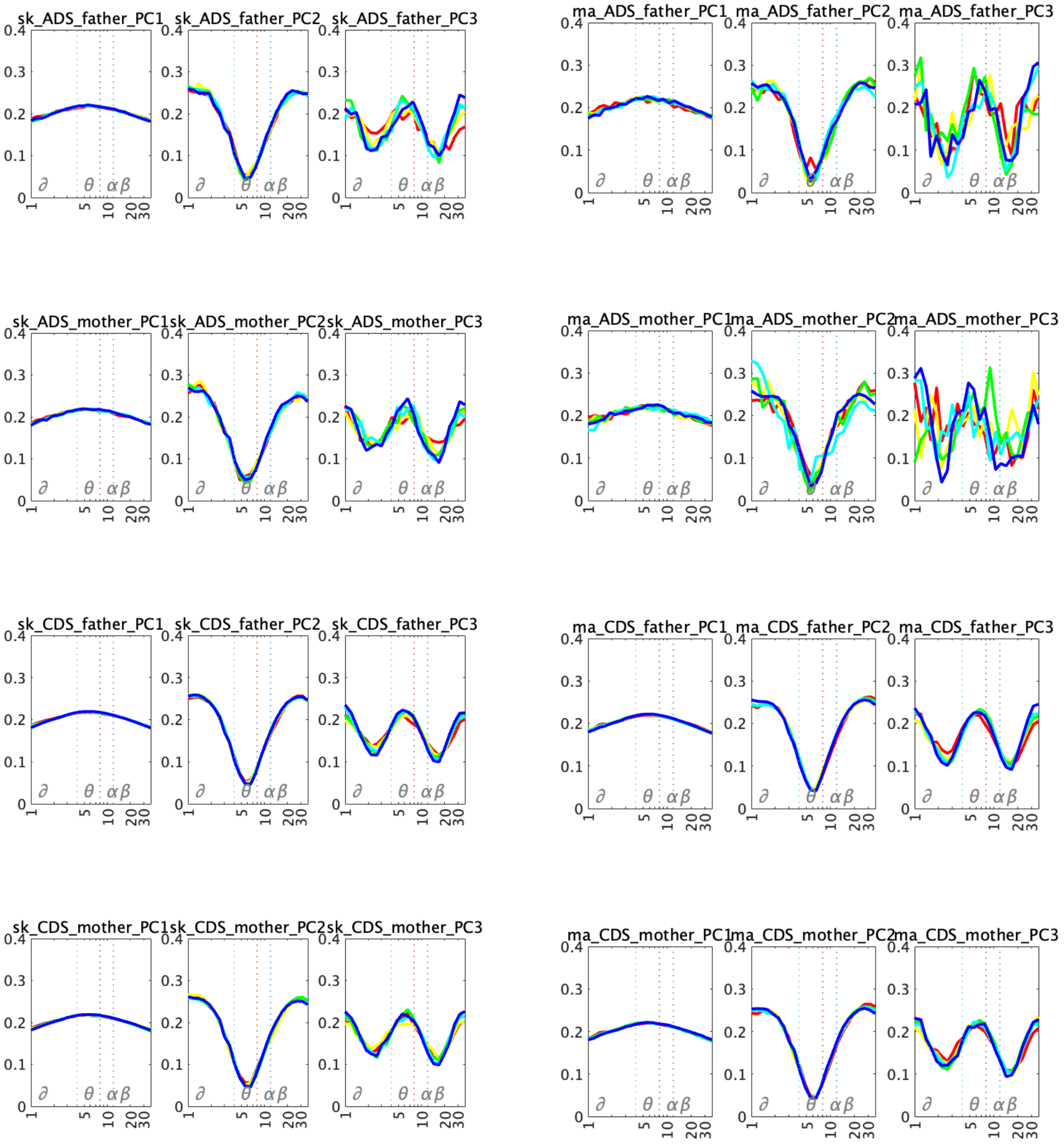
sk_ADS_father_PC1
sk_ADS_father_PC2
sk_ADS_father_PC3
ma_ADS_father_PC1
ma_ADS_father_PC2
ma_ADS_father_PC3
sk_ADS_mother_PC1
sk_ADS_mother_PC2
sk_ADS_mother_PC3
ma_ADS_mother_PC1
ma_ADS_mother_PC2
ma_ADS_mother_PC3
sk_CDS_father_PC1
sk_CDS_father_PC2
sk_CDS_father_PC3
ma_CDS_father_PC1
ma_CDS_father_PC2
ma_CDS_father_PC3
sk_CDS_mother_PC1
sk_CDS_mother_PC2
sk_CDS_mother_PC3
ma_CDS_mother_PC1
ma_CDS_mother_PC2
ma_CDS_mother_PC3
0
0.1
0.2
0.3
0.4
∂
θ
α β
1
5
10
20
30

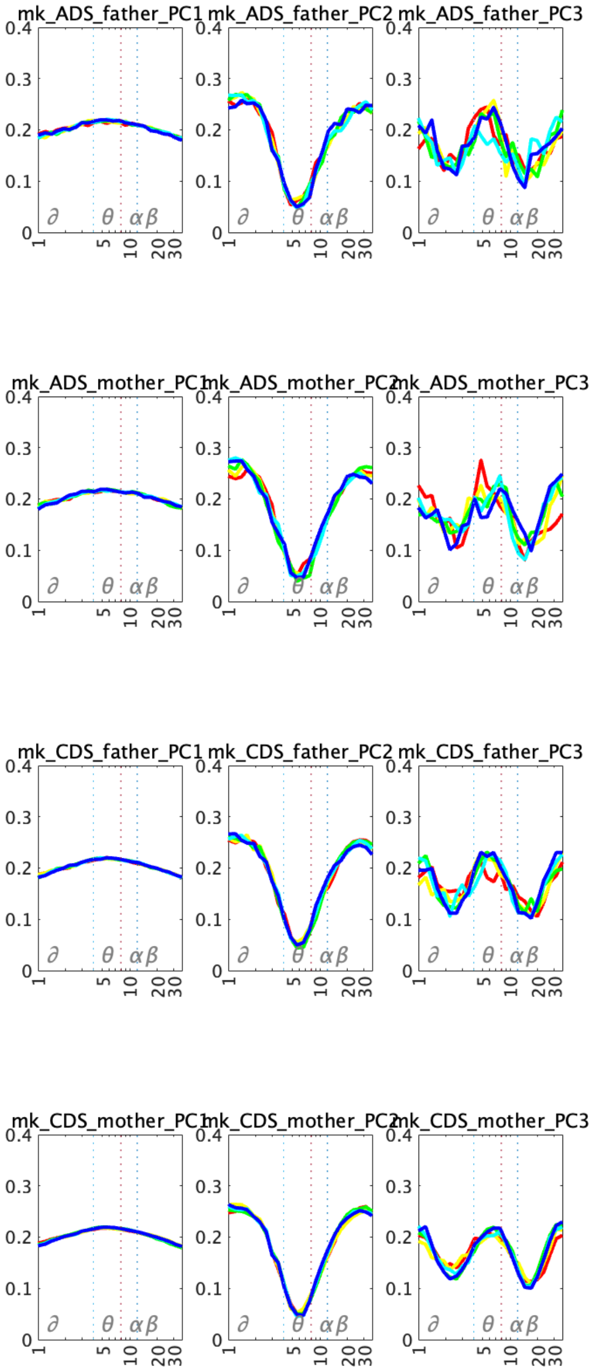
mk_ADS_father_PC1
mk_ADS_father_PC2
mk_ADS_father_PC3
mk_ADS_mother_PC1
mk_ADS_mother_PC2
mk_ADS_mother_PC3
mk_CDS_father_PC1
mk_CDS_father_PC2
mk_CDS_father_PC3
mk_CDS_mother_PC1
mk_CDS_mother_PC2
mk_CDS_mother_PC3

Figure b shows the temporal PCA component loading patterns for each of five families (family ID = sa, kk, sk, ma, mk). In each subplot, the lines of different thickness indicate different PCA components. More important (lower-numbered) components with larger contribution rates are shown in a thicker line, and the components from the spectral bands are plotted in different colours. It may be observed that they produced consistent loading patterns. We, however, also find the individual variation at each temporal modulation band. Because of the consistency and similarity of the loading patterns across participants, the present study considered the core temporal bands using the grand average PC loading patterns. All of the contribution rates in each component and numbers of PC loading have been deposited to an external source (https://osf.io/n3upf/).

Table c. Summary of the 3 temporal bands and the 2 flanking boundaries indentified from temporal PCA.

Temporal bands	Frequency range (Hz)	PC Peaks
Band 1	0.9-2.5	PC2, PC3 in spectral band 1-5
Band 2	2.5-17	PC1, PC3 in spectral band 1-5
Band 3	17-40	PC2, PC3 in spectral band 1-5

Table a: Properties of 5 x 3 Envelopes by S-AMPH

Mean Power

Grand average among families

		band1	band2	band3	band4	band5	average
**Prosody**	**ADS father**	0.00095883(±7.3658e-05)	0.00072002(±4.3016e-05)	0.00010622(±1.1867e-05)	4.636e-05(±3.8929e-06)	4.8943e-05(±4.9676e-06)	0.00037607(±1.3287e-05)
	**ADS mother**	0.0010454(±0.00012979)	0.00081807(±6.1518e-05)	0.00016055(±1.9896e-05)	4.7862e-05(±5.6833e-06)	6.1566e-05(±7.0688e-06)	0.00042668(±2.3048e-05)
	**CDS father**	0.0016414(±6.536e-05)	0.0007741(±2.4501e-05)	0.00013013(±4.4667e-06)	5.2828e-05(±2.7161e-06)	5.4403e-05(±2.5823e-06)	0.00053057(±1.1377e-05)
	**CDS mother**	0.0017014(±8.5213e-05)	0.00090443(±3.4528e-05)	0.00016591(±7.6834e-06)	5.3856e-05(±3.1876e-06)	5.3139e-05(±2.6191e-06)	0.00057574(±1.6014e-05)
**Syllable**	**ADS father**	0.0012383(±8.513e-05)	0.0016093(±0.00011267)	0.00017219(±1.2716e-05)	7.8603e-05(±6.3134e-06)	9.8682e-05(±1.0321e-05)	0.00063941(±2.0731e-05)
	**ADS mother**	0.001486(±0.00016649)	0.0019314(±0.00014943)	0.00024743(±2.3154e-05)	8.5096e-05(±7.6718e-06)	0.00011252(±1.2973e-05)	0.00077249(±2.9354e-05)
	**CDS father**	0.0016826(±6.7752e-05)	0.0014107(±4.9268e-05)	0.00020343(±5.4199e-06)	8.2411e-05(±2.9701e-06)	0.00010304(±3.7318e-06)	0.00069645(±1.1564e-05)
	**CDS mother**	0.0018499(±7.9704e-05)	0.001913(±6.2632e-05)	0.00027507(±1.1727e-05)	8.4336e-05(±3.9796e-06)	9.3658e-05(±4.3659e-06)	0.00084318(±1.5284e-05)
**Phoneme**	**ADS father**	0.00013848(±8.6565e-06)	0.00018595(±1.2927e-05)	2.7477e-05(±2.0479e-06)	1.4596e-05(±1.2345e-06)	1.7239e-05(±1.8515e-06)	7.6749e-05(±2.069e-06)
	**ADS mother**	0.00015888(±1.3953e-05)	0.00023391(±1.7751e-05)	3.4558e-05(±3.5634e-06)	1.5976e-05(±1.4061e-06)	2.051e-05(±2.3225e-06)	9.2768e-05(±3.1254e-06)
	**CDS father**	0.00013912(±3.8223e-06)	0.00013887(±5.4384e-06)	2.6719e-05(±1.0268e-06)	1.3399e-05(±6.0724e-07)	1.6559e-05(±5.5078e-07)	6.6933e-05(±1.1016e-06)
	**CDS mother**	0.00016124(±5.298e-06)	0.00021001(±7.4881e-06)	3.2806e-05(±1.2649e-06)	1.4367e-05(±5.6214e-07)	1.7452e-05(±9.6798e-07)	8.7176e-05(±1.534e-06)

Mean±SEM

** The mean powers in each family have been deposited to an external source (https://osf.io/n3upf/).*

Figure a: Individual Variation in PSI in Each Integer Ratio by S-AMPH model

Grand average of the five families

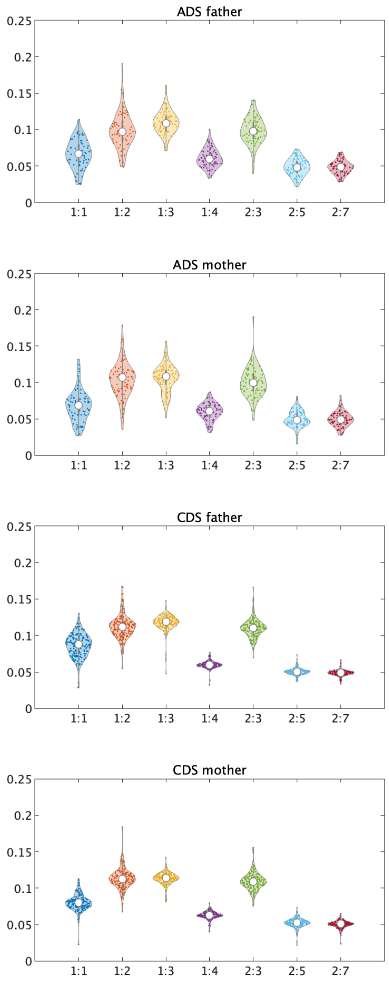

- ADS_father = adult-directed speech by father
- ADS_mother = adult-directed speech by mother
- CDS_father = child-directed speech by father
- CDS_mother =child-directed speech by mother

Each of five families (family ID = sa, kk, sk, ma, mk)

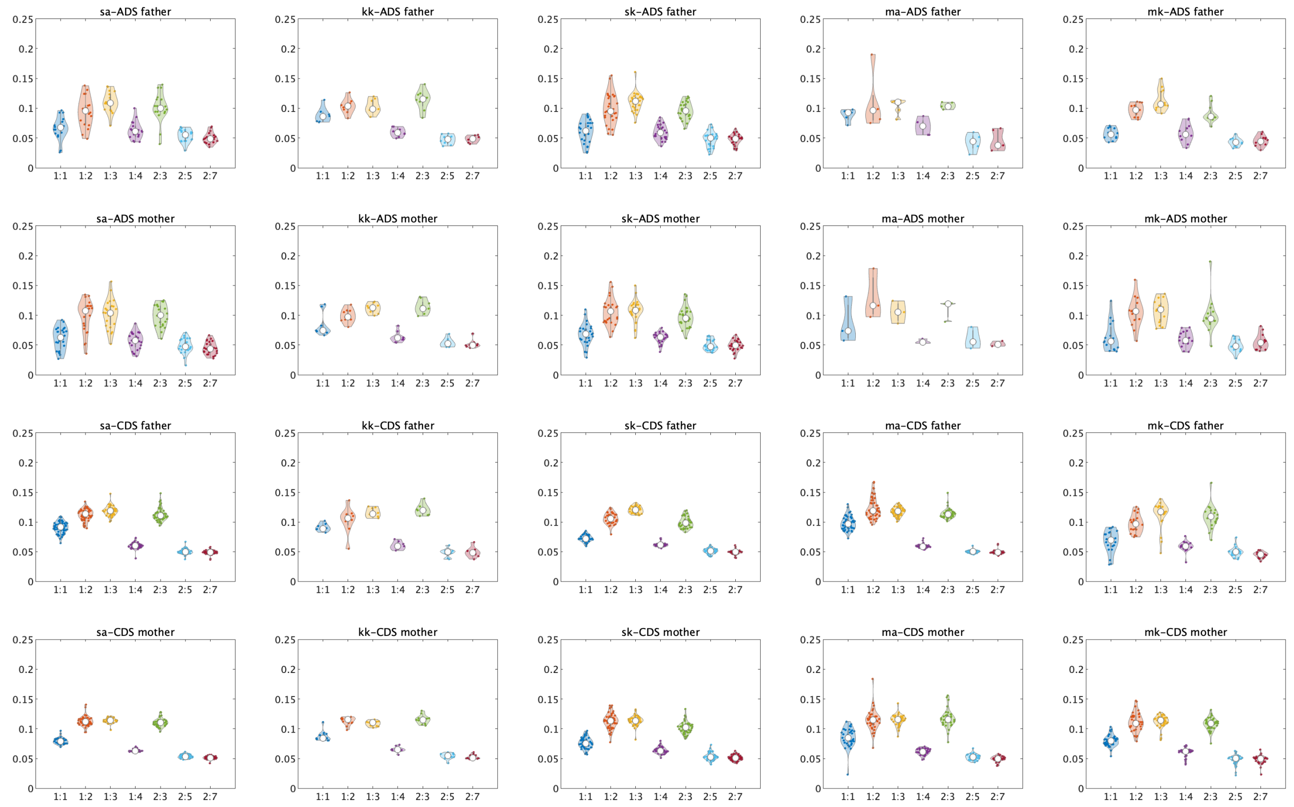

Figure a shows the averaged PSI in each integer ratio by S-AMPH model for each of five families (family ID = sa, kk, sk, ma, mk). In each subplot, the lines of different thickness indicate different types of speech (ADSm, ADSf, CDSf, CDSm). The PSI shown here are broadly similar across the participants. It may be observed that they produced broadly consistent PSIs.

Figure b: Mean delta-theta PSI

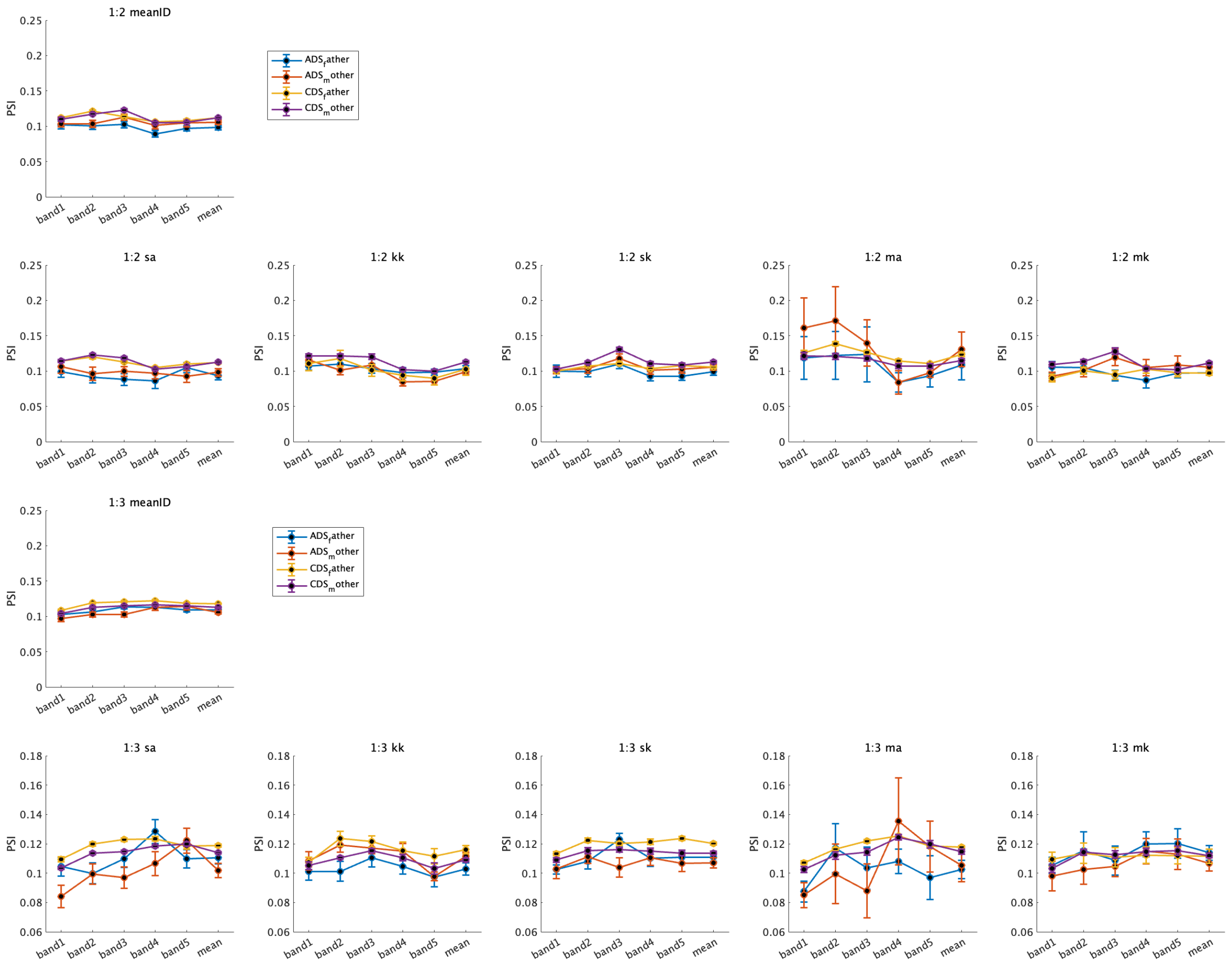

Figure b shows the Phase Synchronization Index (PSI) values based on the average among 1:2 and 1:3 integer ratios and among five families (family ID = sa, kk, sk, ma, mk), for delta-theta AM bands. The x-axis indicates the frequency (spectral) bands. The y-axis shows the PSI value. Error bars indicate the standard error of the mean.

Figure c: Correlation of delta-theta PSI and age

1:2 ratio

Average among five families (i.e., sa, kk, sk, ma, mk)

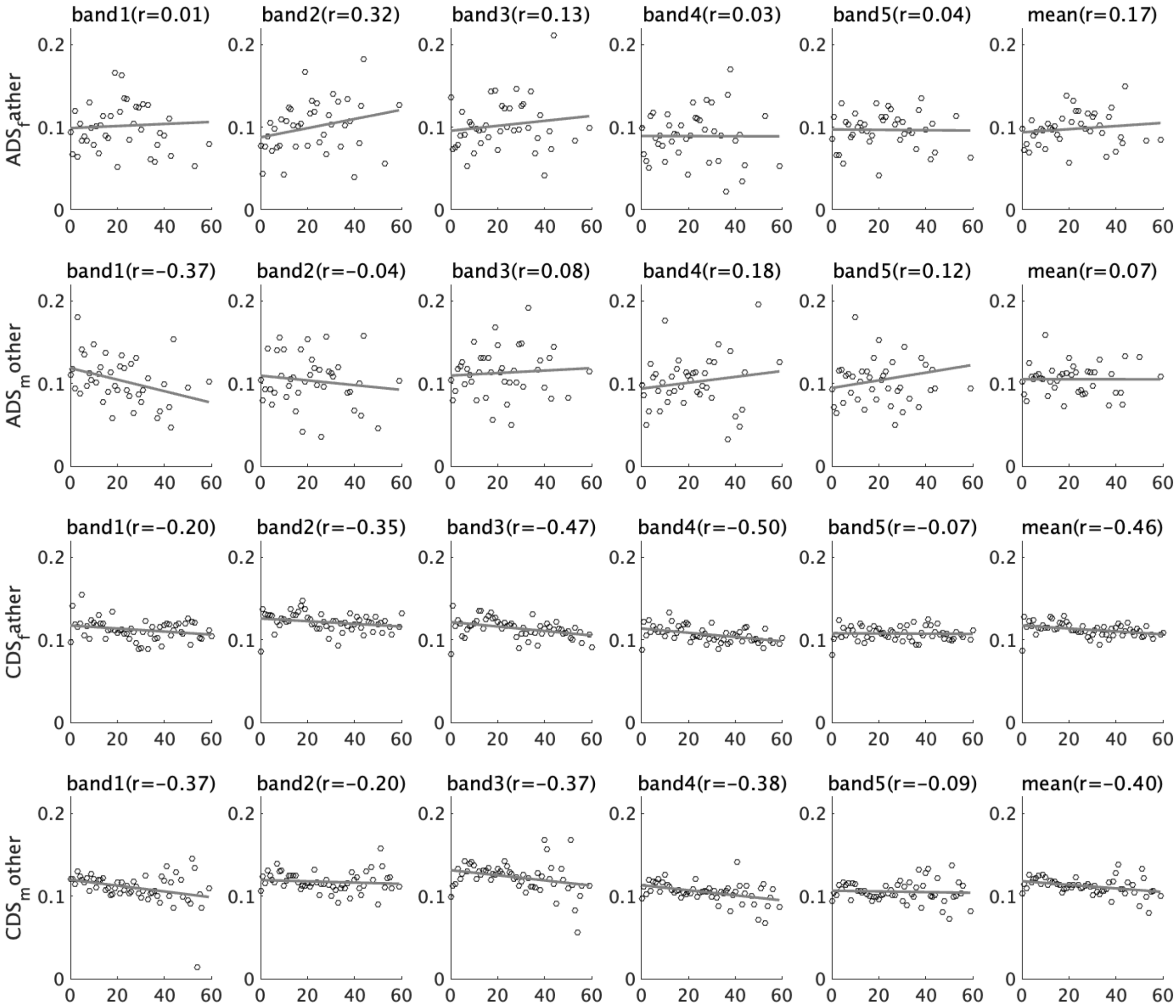

ID = sa

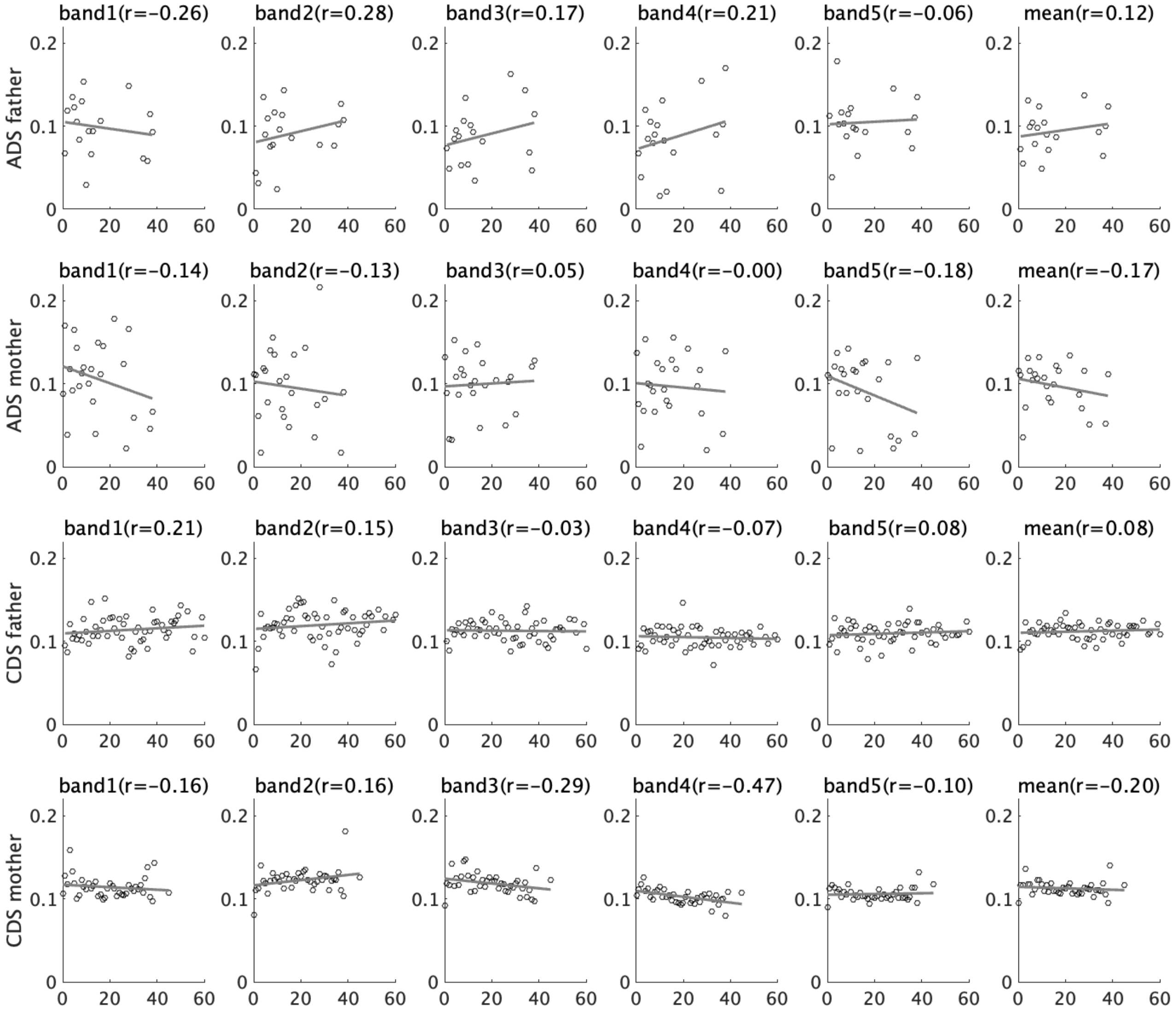
band1(r=−0.26)
band2(r=0.28)
band3(r=0.17)
band4(r=0.21)
band5(r=−0.06)
mean(r=0.12)
ADS father
band1(r=−0.14)
band2(r=−0.13)
band3(r=0.05)
band4(r=−0.00)
band5(r=−0.18)
mean(r=−0.17)
ADS mother
band1(r=0.21)
band2(r=0.15)
band3(r=−0.03)
band4(r=−0.07)
band5(r=0.08)
mean(r=0.08)
CDS father
band1(r=−0.16)
band2(r=0.16)
band3(r=−0.29)
band4(r=−0.47)
band5(r=−0.10)
mean(r=−0.20)
CDS mother
0
0.1
0.2
0
20
40
60

ID = kk

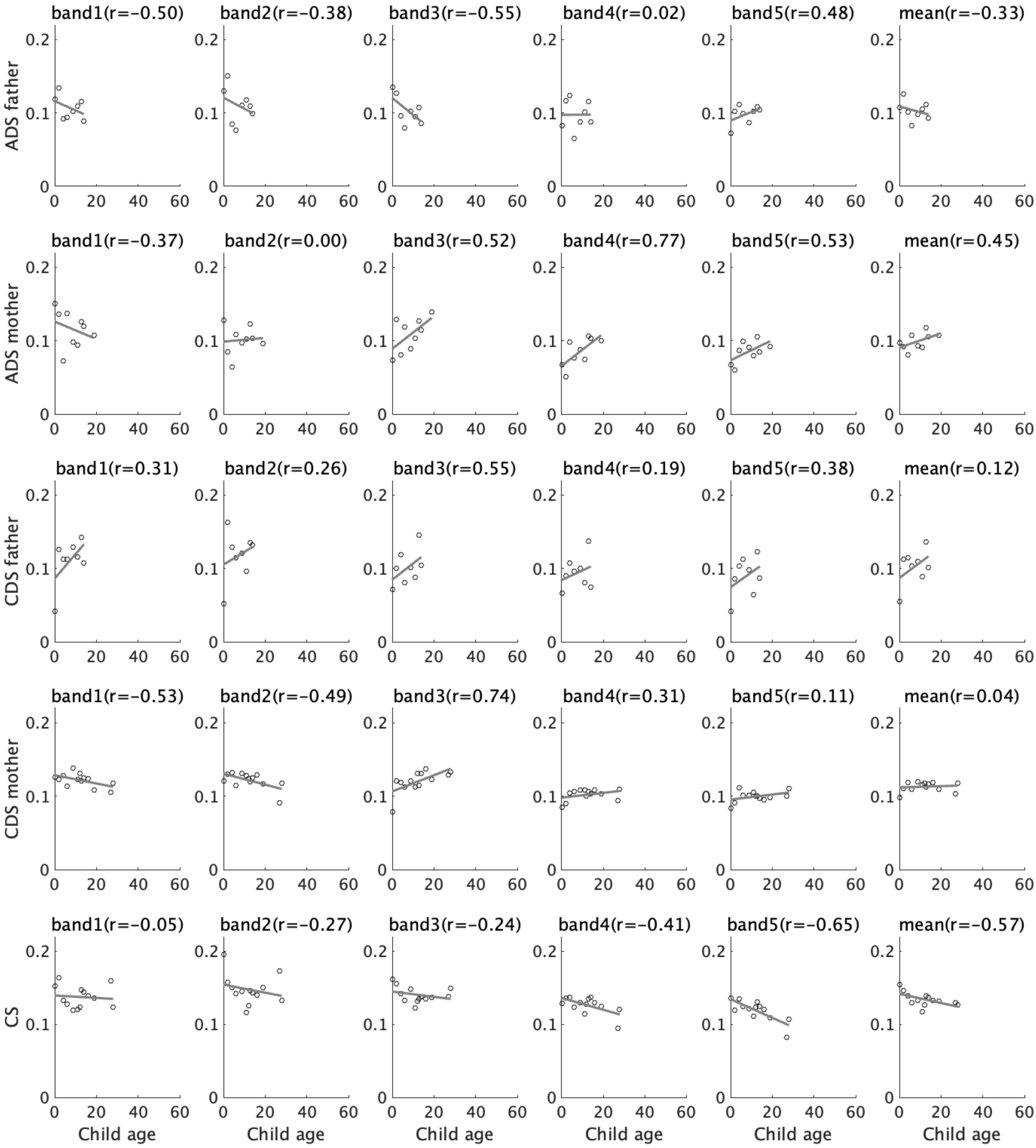
band1(r=−0.50)
band2(r=−0.38)
band3(r=−0.55)
band4(r=0.02)
band5(r=0.48)
mean(r=−0.33)
ADS father
band1(r=−0.37)
band2(r=0.00)
band3(r=0.52)
band4(r=0.77)
band5(r=0.53)
mean(r=0.45)
ADS mother
band1(r=0.31)
band2(r=0.26)
band3(r=0.55)
band4(r=0.19)
band5(r=0.38)
mean(r=0.12)
CDS father
band1(r=−0.53)
band2(r=−0.49)
band3(r=0.74)
band4(r=0.31)
band5(r=0.11)
mean(r=0.04)
CDS mother
band1(r=−0.05)
band2(r=−0.27)
band3(r=−0.24)
band4(r=−0.41)
band5(r=−0.65)
mean(r=−0.57)
CS
0
0.1
0.2
0
20
40
60
Child age

ID = sk

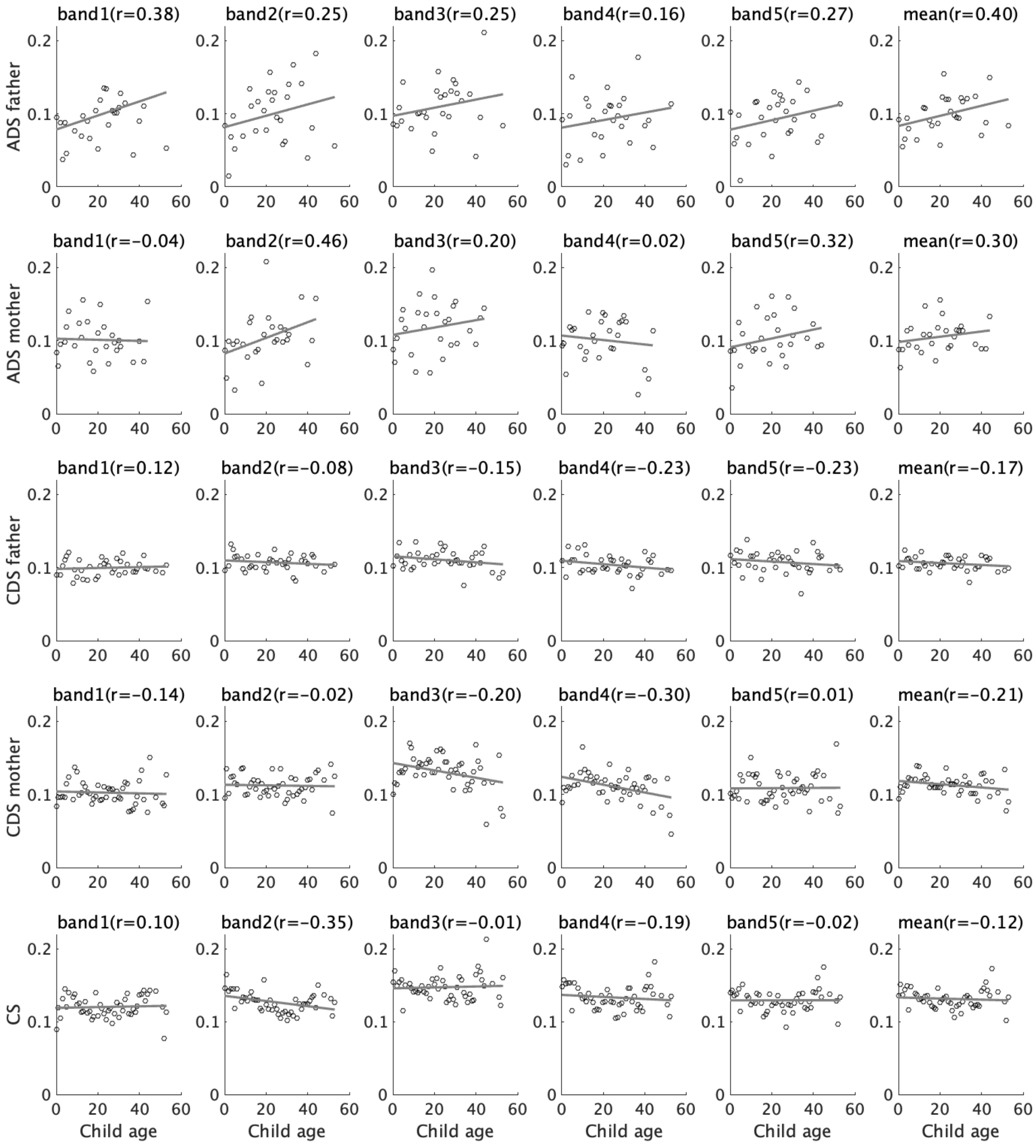
band1(r=0.38)
band2(r=0.25)
band3(r=0.25)
band4(r=0.16)
band5(r=0.27)
mean(r=0.40)
ADS father
band1(r=−0.04)
band2(r=0.46)
band3(r=0.20)
band4(r=0.02)
band5(r=0.32)
mean(r=0.30)
ADS mother
band1(r=0.12)
band2(r=−0.08)
band3(r=−0.15)
band4(r=−0.23)
band5(r=−0.23)
mean(r=−0.17)
CDS father
band1(r=−0.14)
band2(r=−0.02)
band3(r=−0.20)
band4(r=−0.30)
band5(r=0.01)
mean(r=−0.21)
CDS mother
band1(r=0.10)
band2(r=−0.35)
band3(r=−0.01)
band4(r=−0.19)
band5(r=−0.02)
mean(r=−0.12)
CS
Child age

ID = ma

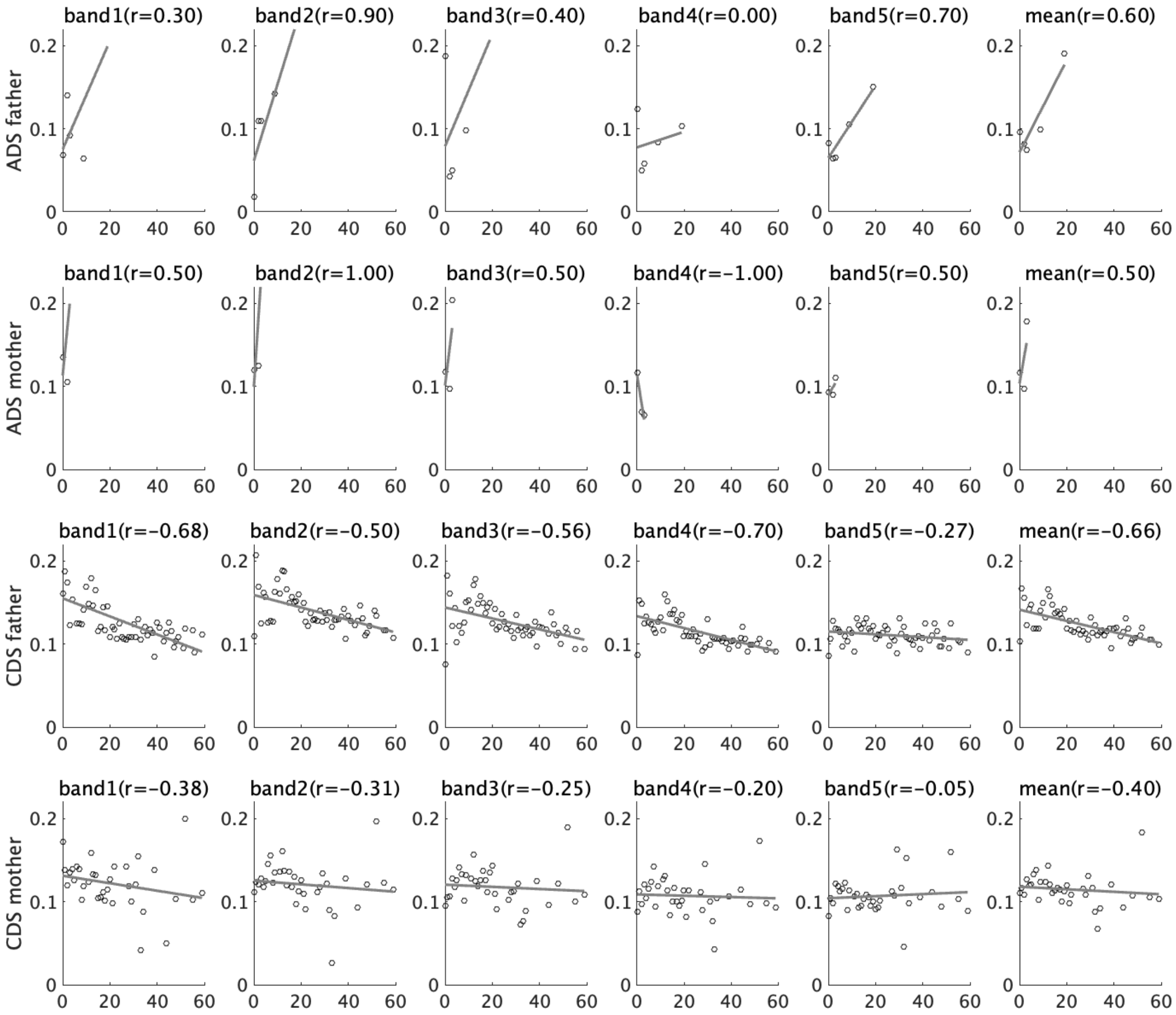
band1(r=0.30)
band2(r=0.90)
band3(r=0.40)
band4(r=0.00)
band5(r=0.70)
mean(r=0.60)
ADS father
band1(r=0.50)
band2(r=1.00)
band3(r=0.50)
band4(r=−1.00)
band5(r=0.50)
mean(r=0.50)
ADS mother
band1(r=−0.68)
band2(r=−0.50)
band3(r=−0.56)
band4(r=−0.70)
band5(r=−0.27)
mean(r=−0.66)
CDS father
band1(r=−0.38)
band2(r=−0.31)
band3(r=−0.25)
band4(r=−0.20)
band5(r=−0.05)
mean(r=−0.40)
CDS mother

ID = mk

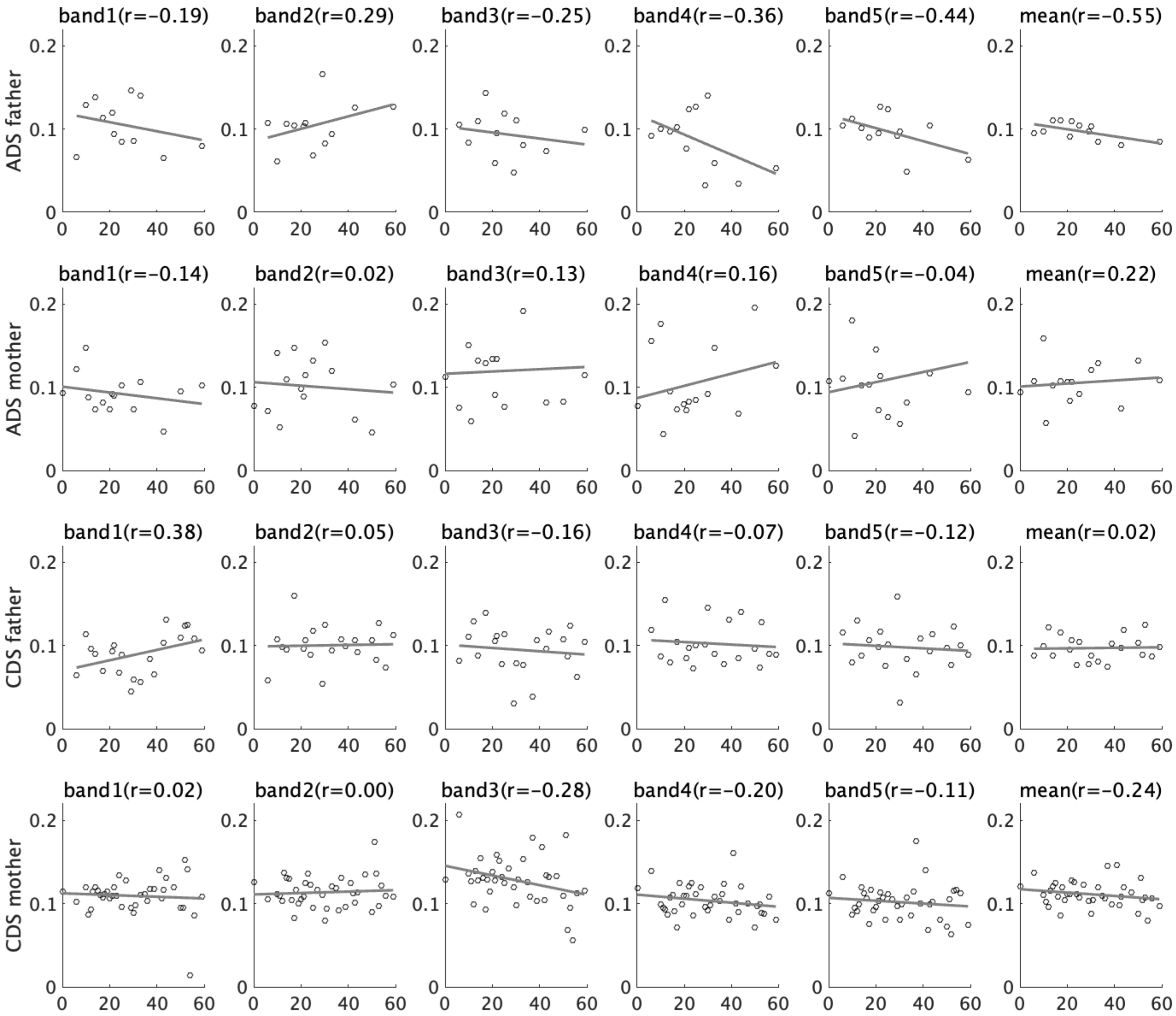

band1(r=-0.19)
band2(r=0.29)
band3(r=-0.25)
band4(r=-0.36)
band5(r=-0.44)
mean(r=-0.55)
ADS father
band1(r=-0.14)
band2(r=0.02)
band3(r=0.13)
band4(r=0.16)
band5(r=-0.04)
mean(r=0.22)
ADS mother
band1(r=0.38)
band2(r=0.05)
band3(r=-0.16)
band4(r=-0.07)
band5(r=-0.12)
mean(r=0.02)
CDS father
band1(r=0.02)
band2(r=0.00)
band3(r=-0.28)
band4(r=-0.20)
band5(r=-0.11)
mean(r=-0.24)
CDS mother
0
0.1
0.2
0
20
40
60

1:3 ratio

Average among five families (i.e., sa, kk, sk, ma, mk)

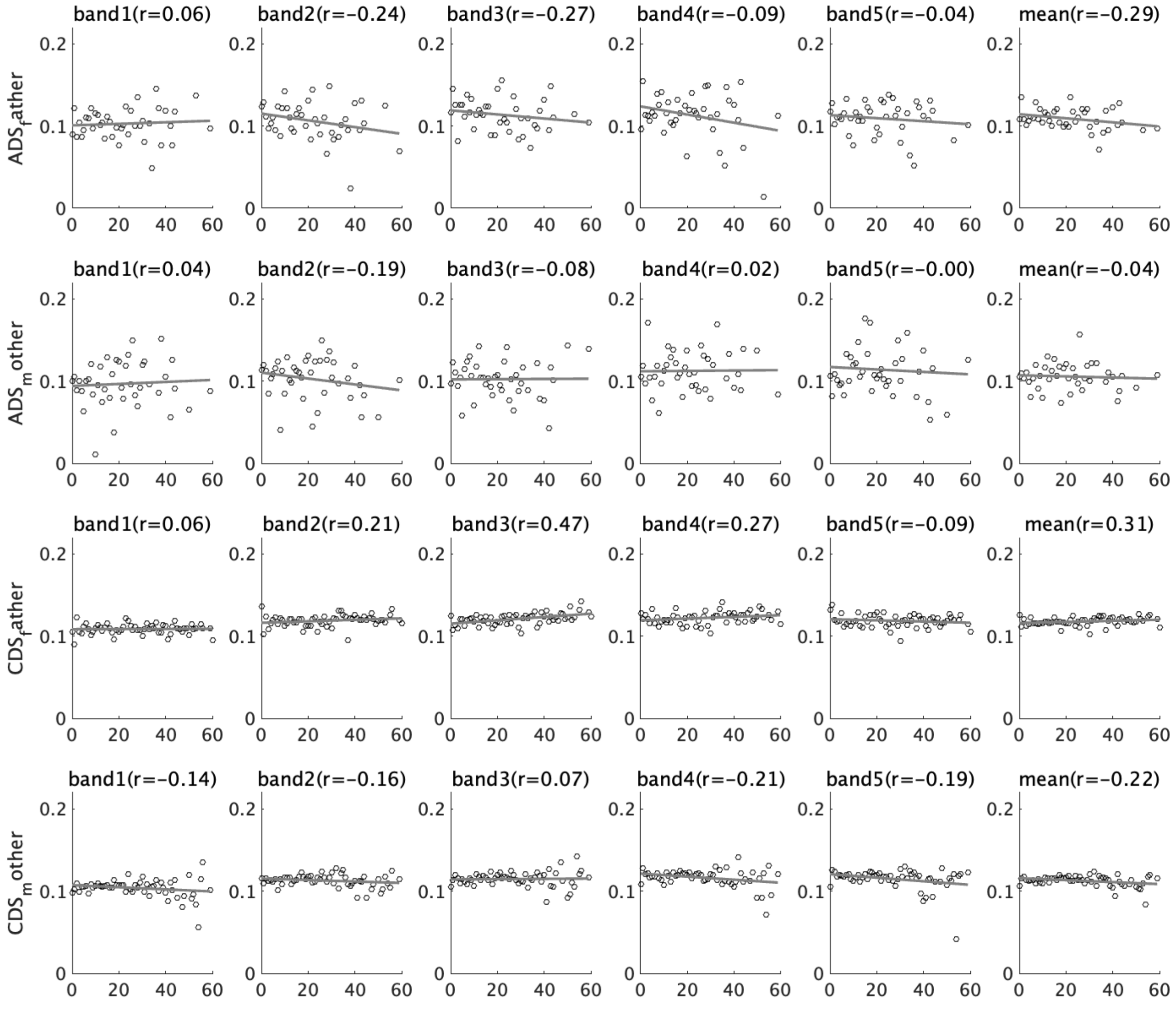

ID = sa

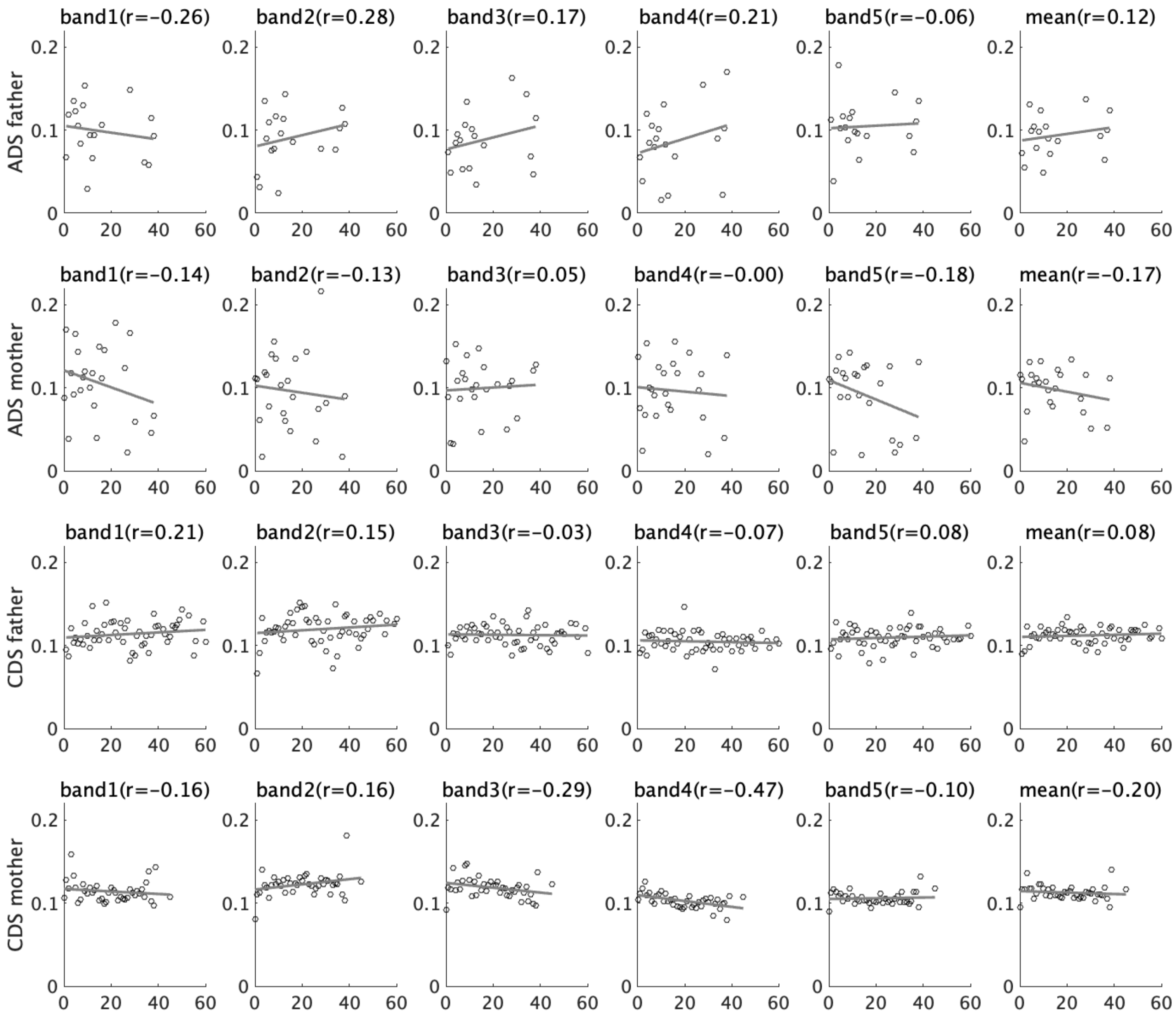

band1(r=−0.26)
band2(r=0.28)
band3(r=0.17)
band4(r=0.21)
band5(r=−0.06)
mean(r=0.12)
ADS father
band1(r=−0.14)
band2(r=−0.13)
band3(r=0.05)
band4(r=−0.00)
band5(r=−0.18)
mean(r=−0.17)
ADS mother
band1(r=0.21)
band2(r=0.15)
band3(r=−0.03)
band4(r=−0.07)
band5(r=0.08)
mean(r=0.08)
CDS father
band1(r=−0.16)
band2(r=0.16)
band3(r=−0.29)
band4(r=−0.47)
band5(r=−0.10)
mean(r=−0.20)
CDS mother
0
0.1
0.2
0
20
40
60

ID = kk

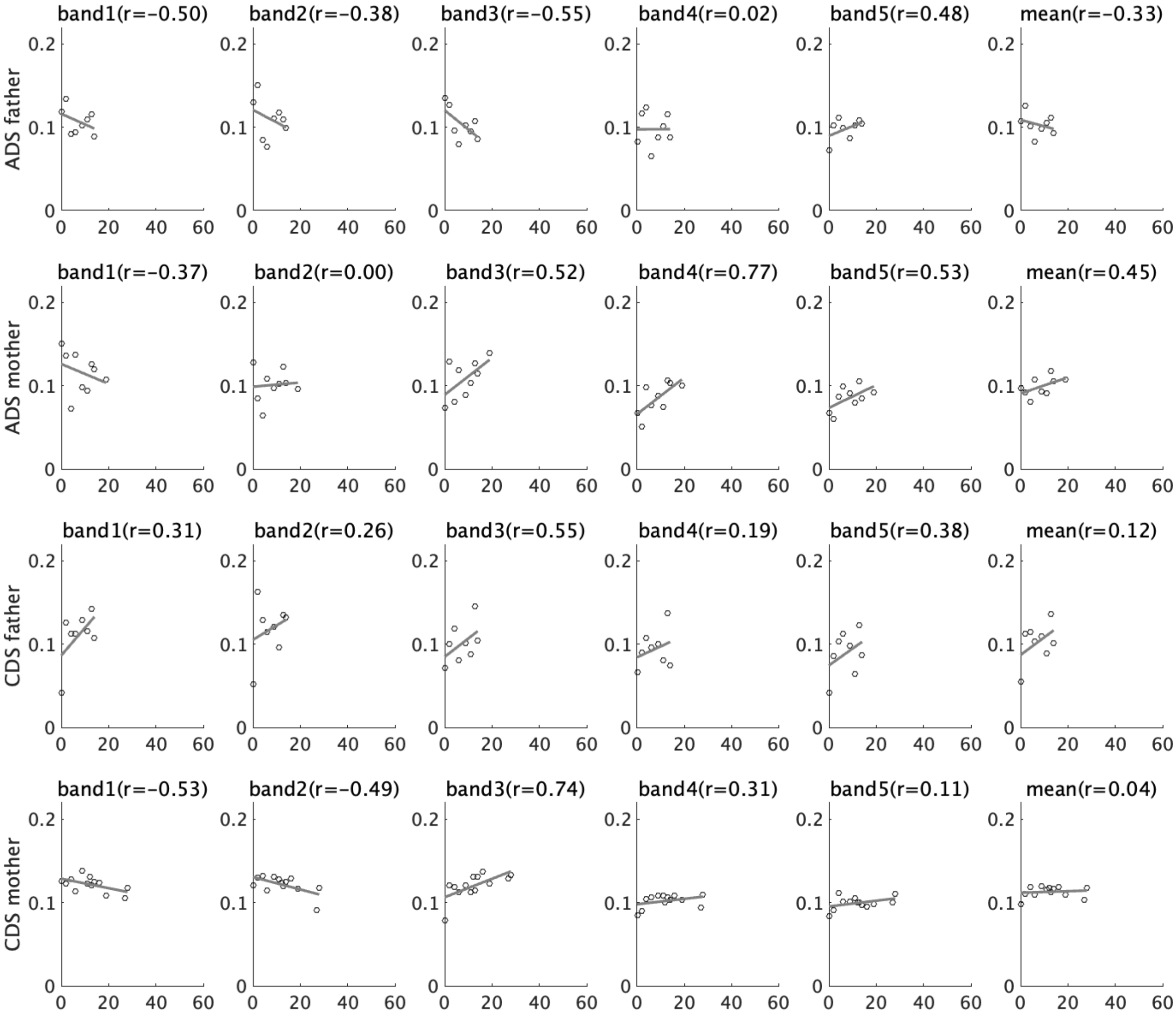
band1(r=−0.50)
band2(r=−0.38)
band3(r=−0.55)
band4(r=0.02)
band5(r=0.48)
mean(r=−0.33)
ADS father
band1(r=−0.37)
band2(r=0.00)
band3(r=0.52)
band4(r=0.77)
band5(r=0.53)
mean(r=0.45)
ADS mother
band1(r=0.31)
band2(r=0.26)
band3(r=0.55)
band4(r=0.19)
band5(r=0.38)
mean(r=0.12)
CDS father
band1(r=−0.53)
band2(r=−0.49)
band3(r=0.74)
band4(r=0.31)
band5(r=0.11)
mean(r=0.04)
CDS mother
0
0.1
0.2
0
20
40
60

ID = sk

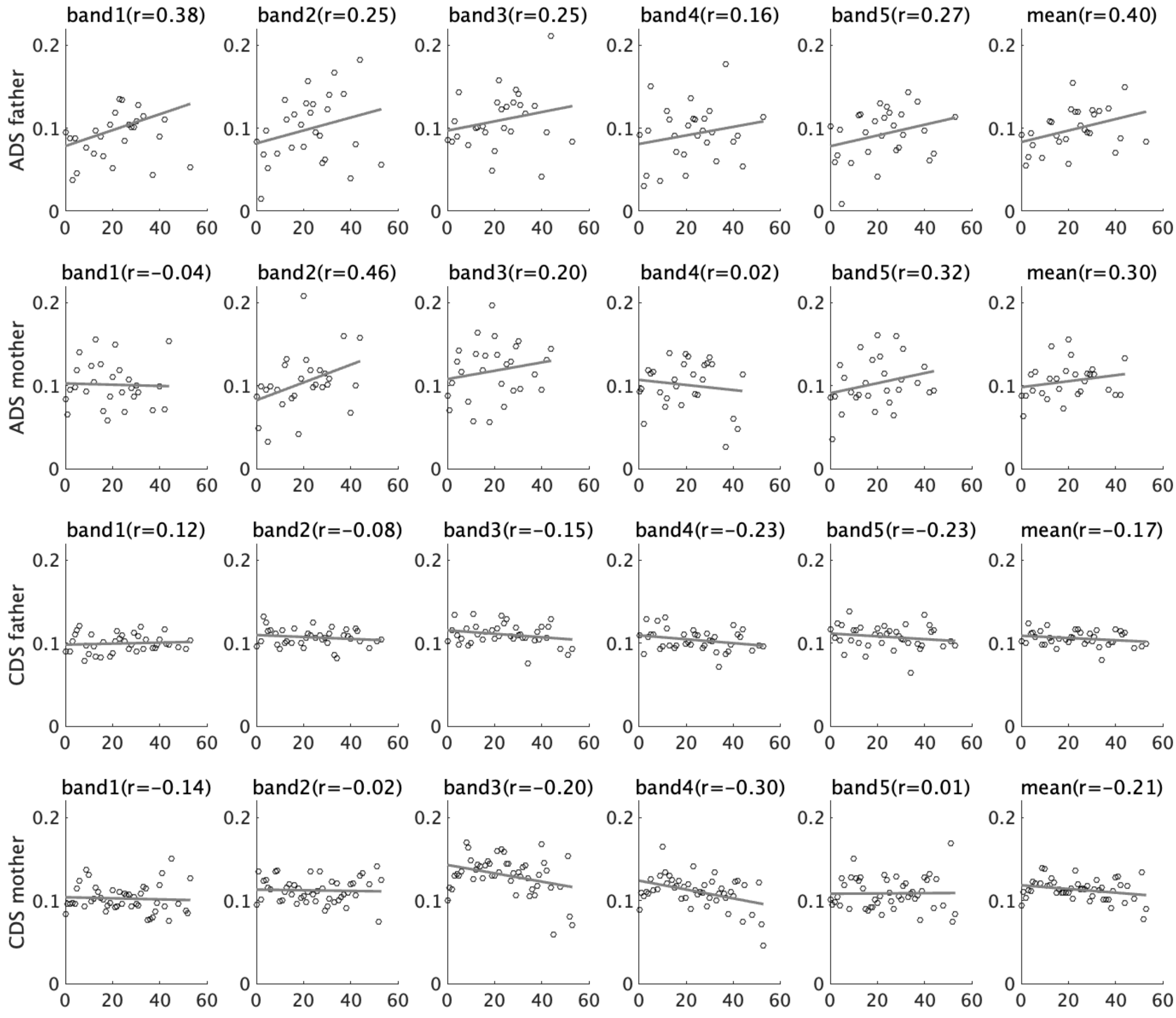
band1(r=0.38)
band2(r=0.25)
band3(r=0.25)
band4(r=0.16)
band5(r=0.27)
mean(r=0.40)
ADS father
band1(r=−0.04)
band2(r=0.46)
band3(r=0.20)
band4(r=0.02)
band5(r=0.32)
mean(r=0.30)
ADS mother
band1(r=0.12)
band2(r=−0.08)
band3(r=−0.15)
band4(r=−0.23)
band5(r=−0.23)
mean(r=−0.17)
CDS father
band1(r=−0.14)
band2(r=−0.02)
band3(r=−0.20)
band4(r=−0.30)
band5(r=0.01)
mean(r=−0.21)
CDS mother
0
0.1
0.2
0
20
40
60

ID = ma

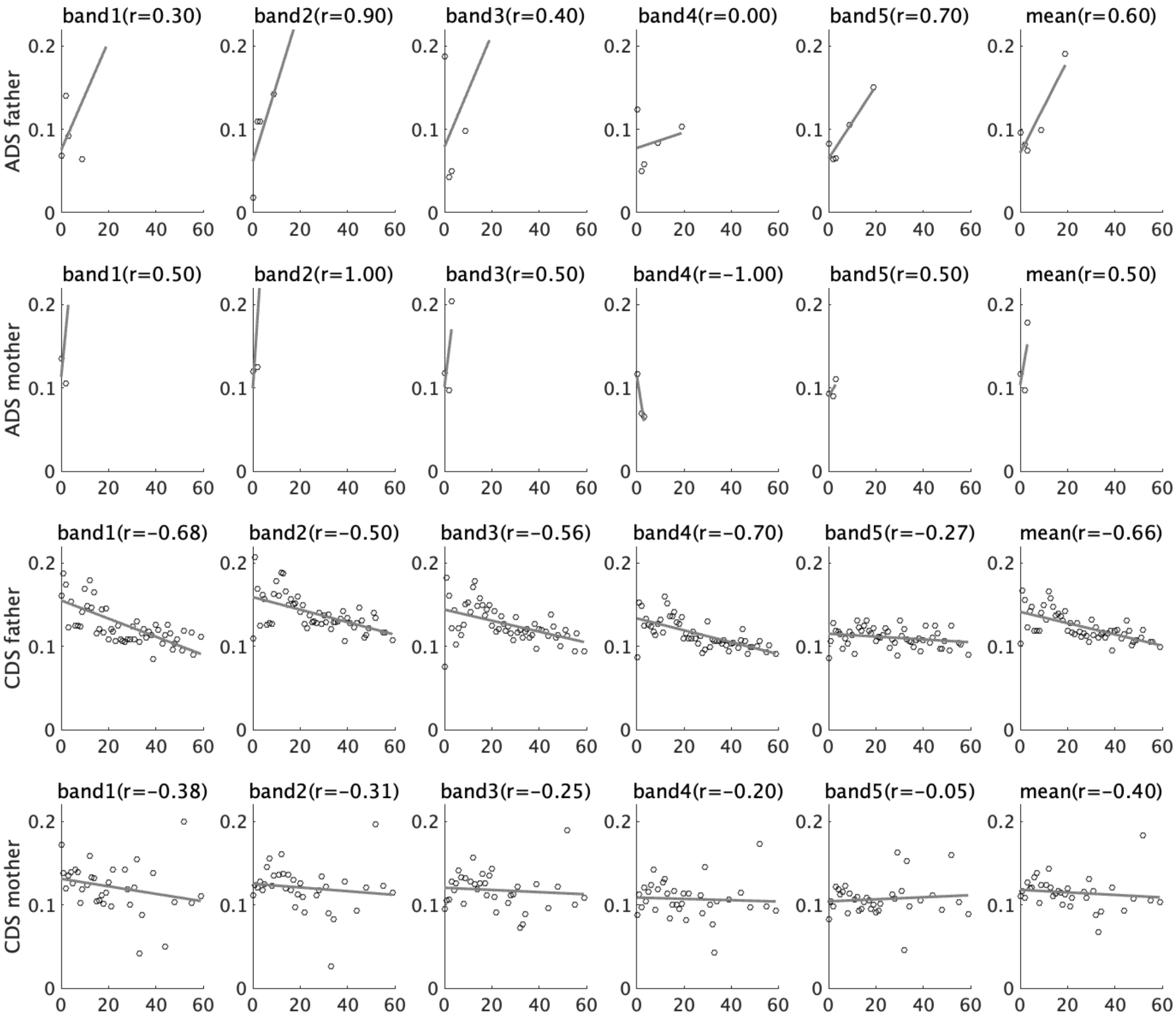

band1(r=0.30)
band2(r=0.90)
band3(r=0.40)
band4(r=0.00)
band5(r=0.70)
mean(r=0.60)
ADS father
band1(r=0.50)
band2(r=1.00)
band3(r=0.50)
band4(r=−1.00)
band5(r=0.50)
mean(r=0.50)
ADS mother
band1(r=−0.68)
band2(r=−0.50)
band3(r=−0.56)
band4(r=−0.70)
band5(r=−0.27)
mean(r=−0.66)
CDS father
band1(r=−0.38)
band2(r=−0.31)
band3(r=−0.25)
band4(r=−0.20)
band5(r=−0.05)
mean(r=−0.40)
CDS mother

ID = mk

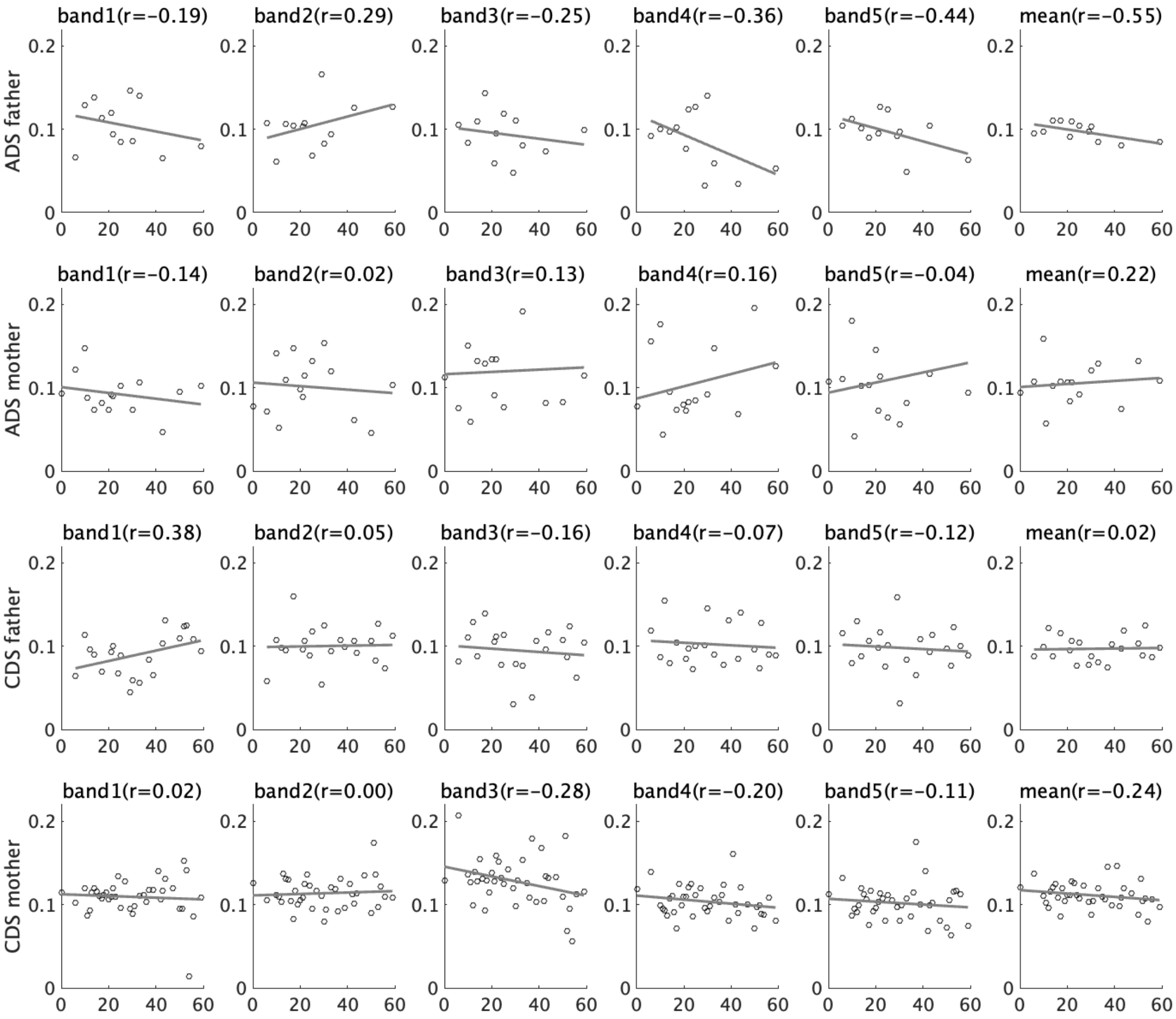
band1(r=−0.19)
band2(r=0.29)
band3(r=−0.25)
band4(r=−0.36)
band5(r=−0.44)
mean(r=−0.55)
ADS father
band1(r=−0.14)
band2(r=0.02)
band3(r=0.13)
band4(r=0.16)
band5(r=−0.04)
mean(r=0.22)
ADS mother
band1(r=0.38)
band2(r=0.05)
band3(r=−0.16)
band4(r=−0.07)
band5(r=−0.12)
mean(r=0.02)
CDS father
band1(r=0.02)
band2(r=0.00)
band3(r=−0.28)
band4(r=−0.20)
band5(r=−0.11)
mean(r=−0.24)
CDS mother
0
0.1
0.2
0
20
40
60
